# Reductive Aromatization of 5,7,12,14‐Pentacenetetrone: Approach Towards Substituted Pentacenes?

**DOI:** 10.1002/chem.202403929

**Published:** 2024-12-30

**Authors:** Olaf A. Kleykamp, Eugen Sharikow, Andreas Stoy, Xiulan Xie, Crispin Lichtenberg, Jörg Sundermeyer

**Affiliations:** ^1^ Department of Chemistry Philipps Universität Marburg Hans-Meerwein-Straße 4 35032 Marburg Germany

**Keywords:** pentacene, cyclic voltammetry, crystallography, density functional theory, reductive aromatization

## Abstract

Herein we report a convenient access to asymmetrically substituted, well soluble pentacene derivatives synthesized from commercially available 5,7,12,14‐pentacenetetrone *via* reductive one step functionalization. Zinc or potassium are used as reducing agents and the reduced intermediates are trapped with electrophiles such as acetyl, tri*iso*propylsilyl or cationic methyl synthons. The reduction allows for an unsymmetric functionalization whilst one dione moiety is maintained due to partial reduction. The resulting dyes are characterized by UV/Vis and photoluminescence spectroscopy. Their redox potentials are studied by cyclic voltammetry. The experimentally determined molecular frontier orbital energies are rationalized by density functional theory. The spatial molecular packing motive in the solid state is studied as a function of the substituent size by X‐ray crystallography. Attempts of reducing the second quinone moiety towards a functionalized pentacene led to an over‐reduced 6,13‐dihydropentacene derivative, which by means of an XRD structural analysis surprisingly displays a planar molecular conformation and unique optoelectronic properties.

## Graphical abstract

5,7,12,14‐Pentacenetetrone was functionalized *via* reductive aromatization strategy. The products were characterized by UV/Vis, CV, XRD, and discussed in relation to DFT methods. This method tends to yield partially reduced or over‐reduced pentacene derivatives.

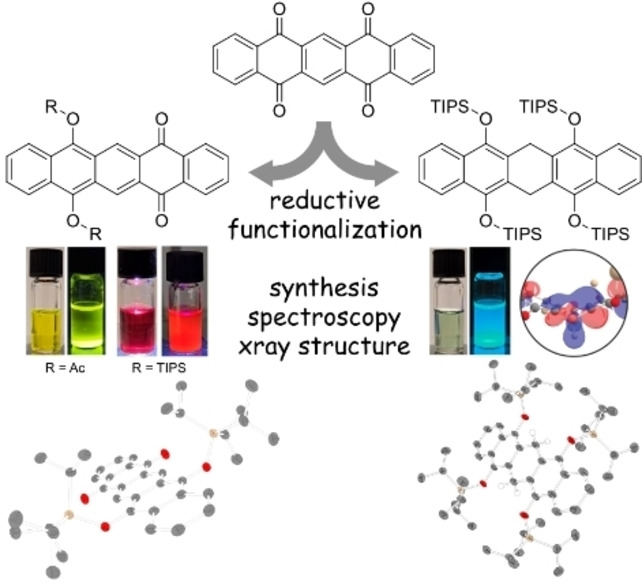

## Introduction

**Scheme 1:**
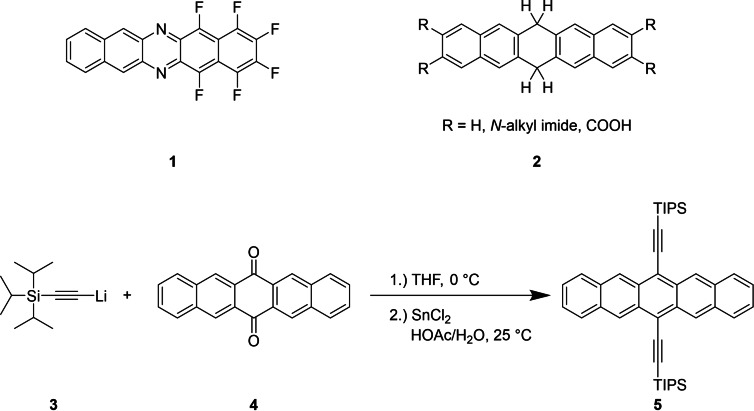
Representative centrally aza‐substituted pentacene derivative **1** ^[8]^ and related 6,13‐dihydropentacenes **2**[^13^, ^14^, ^15^]. Common approach to stabilized pentacene derivatives **5** *via* dione **4**, addition of organolithium reagents and reduction of the alcohol functionality *via* tin(II) chloride.^[16]^

Polyaromatic hydrocarbons (PAH) became a field of high interest due to their broad application potential: Historically, PAHs have been examined as dyes, their optical properties have been investigated and during this research it has been found that optical and electronical properties are coherent.[^1^, ^2^] Conductive organic polymers were discovered in the 1970’s and the science community witnessed the rise of a new class of semi‐conductive materials.^[3]^ In contrast to organic polymers, which have a better processability in solution, single molecule PAHs show higher electron mobility, especially in lattice optimized reassembled arrays.^[4]^ The aromatic systems can be expanded either laterally (acenes) or in peri position (rylenes). Perylenes provide outstanding chemical, thermal and photochemical inertness in combination with a low band gap and NIR absorption and emission.^[5]^ In contrast to rylenes, acenes provide an all zig zag edge structure and the aromatic system can be extended in lateral dimension. Following this strategy, the HOMO‐LUMO energy gap is narrowed, and an open‐shell ground state character is generated in the higher homologues.^[6]^ Pentacene with its five annulated benzene rings is the smallest homolog showing a diradical character with a high spin density being located in the central benzene ring which makes the molecule prone to oxidation with ^3^O_2_ to form an endo epoxide.^[7]^ This oxidative transformation can be hindered by substitution of the central carbon atoms by nitrogen (Scheme 1, **1**).^[8]^ Due to this intrinsic instability and high reactivity, the synthesis of parent higher acenes in solution is challenging. Introducing large trialkylsilylethynyl groups provide improved solubility and stability in the case of nonacene as the longest acene synthesized in solution.^[9]^ Due to their planarity, acenes tend to crystallize easily and typically provide highly ordered lattice structures. Those packing motives can have an extensive influence on the electronic properties (e. g. singlet fission) in the solid state, which is important for technical applications.^[10]^ Because of the tunable properties of the solid state by synthetic modifications, they have frequently been used for organic semiconductors (OSCs), organic field effect transistors (OFETs), organic light emitting diodes (OLEDs), organic light emitting transistors (OLETs) and photovoltaic devices.[^11^, ^12^]

As a result of their unique properties, acenes emerged as a thoroughly investigated class of molecules PAH.[^16^, ^17^, ^18^] Comparatively small anthracene derivatives became a frequently used motive for organic thin‐film semiconductors because of their high charge carrier mobility, narrow HOMO‐LUMO energy gap combined with a good thermal and photochemical stability.^[19]^ By functionalization of the anthracene backbone at different positions with functional groups, the optoelectronic properties as well as their stability and solubility can be optimized and adjusted.^[20]^ These aromatic systems display an ordered packing in solid state, where three types (cofacial, parallel‐displaced and edge to face) are differed. The capability that a charge carrier hops from one to an adjacent molecule is described as transfer integral.^[21]^ It is dependent on the overlapping area of the π‐system and their π‐π‐stacking distance. Displacement over the long and short axis of two stacked molecules lowers the transfer integral.^[22]^ Both, the distance and the displacement, can be influenced by the spatial demand of the functional group.^[23]^ It has previously been shown that altering electron donating with withdrawing moieties influences the energy levels of the frontier molecular orbitals (FMOs). This results in an adjustable energy gap and modifiable UV/Vis absorption properties.^[24]^ DFT calculations are also capable of predicting the single electron redox potentials^[25]^ and FMO energies^[26]^ and TD‐DFT calculations forecast UV/Vis absorption energies.^[27]^ These data derive from the experimental data most of the times, nevertheless they can indicate a trend in FMO and absorption energies.^[28]^

Reductive aromatization has proven as a reliable method to introduce functional groups from quinoid systems^[29]^ and other carbonyl group containing PAHs as starting materials.[^30^, ^31^, ^32^] It is possible to either maintain or remove the oxygen functionality of the backbone. For the latter strategy, the reaction with an organo lithium reagent followed by reduction with tin(II) chloride has been established (Scheme 1, **5**).[^33^, ^34^] Inorganic reducing agents like sodium dithionite (in water), zinc and potassium under anhydrous conditions in combination with the desired electrophiles are commonly used to first reduce the quinone and maintain the oxygen of the backbone.^[35]^ 6,13‐Dihydropentacenes like **2** can be synthesized from the corresponding quinoid systems by acidic reduction, which was shown by Takaguchi.^[13]^ A further approach to tetrasubstituted 6,13‐dihydropentacenes is the nucleophilic addition of a lithium organyl and subsequent methylation of the formed alkoxide.^[36]^ 5,7,12,14‐Pentacenetetrone, the synthon of this study, has been discussed as an additive to lithium ion batteries and has demonstrated the expected four‐electron redox behavior.^[37]^ It was claimed that a larger π‐system in combination with a chemically modified pentacene derivative can enhance the cycle stability of organic electrodes.^[38]^ This study of reductive functionalization focusses on the isolation of derivatives obtained from potassium reduction of this pentacenedione followed by trapping the intermediates by electrophiles.

## Results and Discussion

### Synthesis of Pentacene Derivatives

**Scheme 2:**
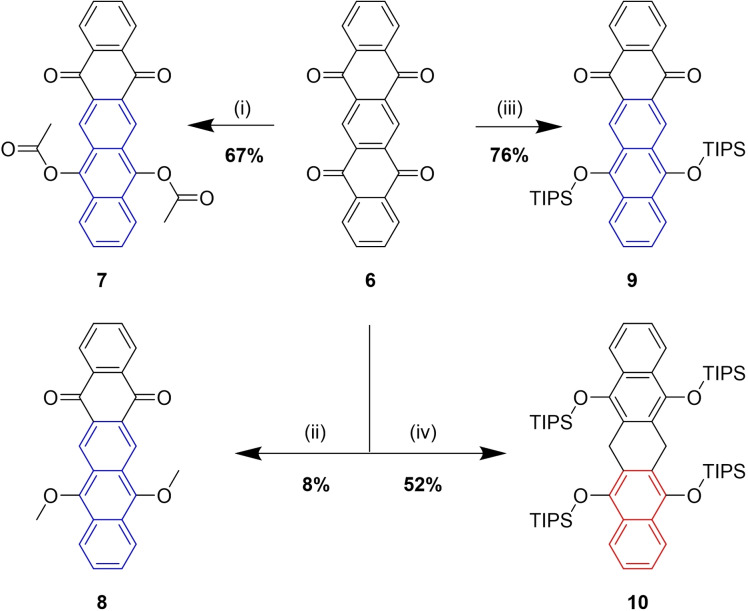
Partial reductive aromatization of tetraone **6** towards anthracene derivatives **7**, **8**, **9** and over reduction to dihydropentacene **10**:

5,7,12,14‐Pentacenetetrone (**6**) (Scheme 2) is commercially available and readily accessible *via* Friedel‐Crafts acylation of benzene and pyromellitic dianhydride.^[39]^ It is poorly soluble in common organic solvents but can be solubilized through reductive functionalization by groups that reduce the π‐stacking by sterical hinderance. Successful attempts to use **6** as a starting point to synthesize functionalized pentacene derivatives were reported by Artamonova *et al*. who obtained partially reduced monoquinone **7**, *via* zinc reduction of **6** in the presence of acetic anhydride.^[40]^

We were not successful in reducing **6** with zinc and quenching the intermediate zinc aryloxides by acetyl chloride in 1,4‐dioxane at 90 °C. No conversion was observed. A former approach of utilizing 5,7,12,14‐pentacenetetrone in reductive functionalization reactions was made by Sekora and Seka in 1927.^[41]^ They reported the formation of a benzoic ester derivative of **7** by reduction with sodium dithionite in an aqueous solution of sodium hydroxide followed by reaction with benzoyl chloride. In our hands following their procedure with acetyl chloride instead did not yield any acetyl derivative **7**. Therefore the potential of stronger reducing metallic potassium was explored in order to selectively reduce one or even the second quinone functionality towards a functionalized pentacene skeleton and to trap the intermediate O‐nucleophiles with other electrophiles, similar to strategies investigated previously in our group.[^24^, ^30^, ^35^, ^42^] When stronger reducing metallic potassium ($\epsilon_{0}^{o}\left(K\right)=<M->2.925\;V\;vs.\;\epsilon_{0}^{o}\left(Zn\right)=<M->0.763\;V$ )^[43]^ was used as a reducing agent, **7** was obtained in good yields of 67 % as yellow needles after filtration and recrystallisation. Aggregation and nucleophilicity of the intermediate metal aryloxy species seem to play an important role. A reductive methylation of **6** to **8** was also realized using metallic potassium (6 eq.) and methyl iodide under the same conditions as described above (Scheme 2(ii)). The reaction mixture was filtered, and **8** was crystallized from *n*‐pentane and dichloromethane as orange crystals, however in poor yield of 8 %. Reactions using KC_8_ as a better chargeable reducing agent turned out to give similar yields compared to metallic potassium and no advantage due to difficulties in separating product from graphite in dioxane. As described previously for quinoid structures^[44]^, the reduction of **6** was carried out using an excess of zinc in combination with tri*iso*propylsilyl chloride (TIPS‐Cl) and imidazole as a nucleophilic catalyst in 1,4‐dioxane at 90 °C overnight, resulting in a red solid with a good yield of 76 %. An excess of metallic potassium (10 eq.) as a more powerful reducing agent and treatment with TIPS‐Cl after full reduction (16 h, 90 °C) of the second quinone functionality did not result in a pentacene but in an unexpected product of overreduction of the central ring. 6,13‐Dihydropentacene derivative **10** was isolated under argon as a green solid in 52 % yield. (Scheme 2 (iv)). The yield of the reaction was optimized starting from 13 % by changing the purification procedure. The first procedure included a simple wash with methanol to remove the excess of TIPS−Cl and a part of the dissolved product, unfortunately. In the optimized work‐up, excess of TIPS−Cl was reacted for 1 h with methanol and volatiles removed under reduced pressure. The resulting green precipitate was washed with *n*‐pentane through a pad of celite. Thereafter, the green solid product was eluted with dichloromethane to obtain **10** in better yield of 52 %. The reactions can be monitored *via* a color change from yellow/brown to a fluorescent, purple mixture and concomitant consumption of the poorly soluble starting material. It seems, that under these reaction conditions, a potentially substituted pentacene intermediate is faster reduced than it might have formed *via* the reduction of the second quinone moiety in the presence of TIPS−Cl. A plausible explanation could be, that pentacene would be stabilized by only one Clar sextet, whereas 6,13‐dihydropentacene **10** is comprising two Clar sextets, one in each naphthalene subunit. ^13^C{^1^H} NMR spectroscopy shows one methylene carbon signal which is in accordance with four chemically equivalent methylene protons seen from ^1^H NMR spectroscopy. In addition, HR‐mass spectroscopy (FD^+^) gives the *m/z* ratio which matches the positively charged 6,13‐dihydropentacene **10**.

The reviewers drew our attention to the synthetic study of Sekora and Seka, who claimed the synthesis of 5,7,12,14‐tetra‐acetoxy pentacene in 1927.^[41]^ Following exactly their procedure of reducing **6** with zinc in presence of anhydrous sodium acetate in acetic anhydride under reflux, we also isolated a yellow solid. According to NMR and HR‐MS studies we conclude that this raw material contains at least two components which could not be separated *via* TLC or column chromatography. The ^1^H‐NMR spectra in dichloromethane‐*d_2_* of this mixture is displayed in Figure S8 – S12. They display a signal set which might be assigned to an asymmetrically substituted dihydropentacene, e. g. 9,13‐diacetoxy‐dihydropentacene. A methylene signal observed at δ=4.15 ppm (25 °C) is showing coalescence at −13 °C and splits into an AB pattern, another methylene signal from a second species is not showing any coalescence. Further purification by preparative HPLC or other techniques and investigation of the products of this pioneering report are ongoing.

**10** turned out to be unstable in chloroform under light and argon indicating that it might be prone towards oxidation. Dihydroanthracenes have already been discussed as hydrogen donor molecules.^[45]^ Attempts to selectively oxidize **10** to a pentacene using DDQ as a stoichiometric dehydrogenation agent lead to a loss of the methylene groups, but also to a mixture of products was formed according to ^1^H, ^13^C and ^29^Si NMR spectra (see SI, Figure S14‐S16). The oxidation of **10** in presence of oxygen did not lead to a decomposition of the starting material. Furthermore, the potential of **10** to act as a hydrogen atom donor was probed by addition of an equimolar amount of 2,2,6,6‐tetramethylpiperidinyloxyl (TEMPO).^[46]^ Hydrogen atom transfer from **10** to TEMPO would lead to a decrease of the TEMPO radical concentration and an increase of a newly formed [**10**‐H] radical species or its disproportionation towards a mixture of pentacene [**10**‐2H] and dihydropentacene **10**. However, the reaction of **10** with TEMPO toluene at room temperature and even at 100 °C did not show any change in the measured EPR spectra and signal intensity. Thus, hydrogen atom abstraction from **10** by TEMPO was not feasible as preparative method under the given reaction conditions. In order to study one‐electron oxidized species of **10** to a radical cation, a titration experiment with nitrosyl tetrafluoroborate (NOBF_4_) was carried out and monitored by UV/Vis absorption. The reaction mixture turned from green to red gradually, starting from addition of 2 eq. of NOBF_4_. It was found that the absorption at 430 nm decreased, whereas the absorption in a range of 460 nm to 560 nm increased. The spectra can be found in the SI (Figure S5). In theory, **10** can be oxidized to the dictation in two steps followed by elimination of two protons which would lead to a pentacene. Unfortunately, the product has not been isolated. A similar reaction of **10** with KC_8_ in THF did not show a similarly strong influence on the absorption properties.

### Crystallography

**Figure 1:**
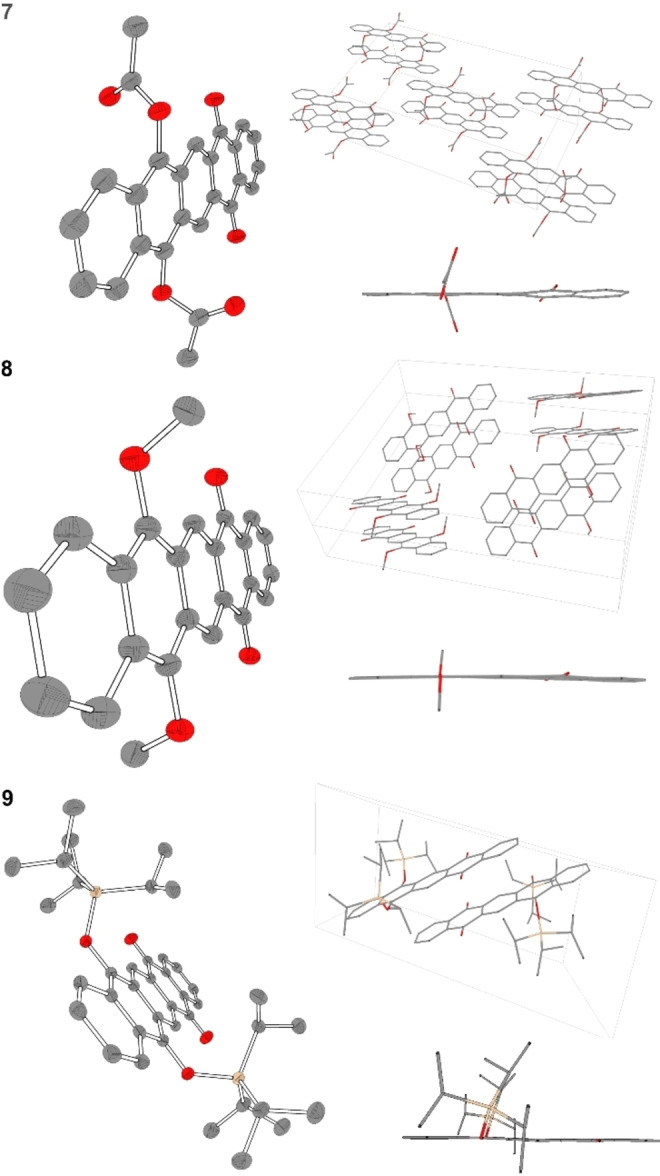
Solid state structures and packing of **7**, **8**, **9**. Hydrogen atoms and solvent molecules are omitted for clarity and thermal ellipsoids are shown at 50 % probability level. π ‐π Distances: **7**: 3.34 Å, **8**: 3.50 Å, **9**: 3.23 Å

X‐ray diffractive single crystals were obtained by gas phase diffusion of *n*‐pentane in a saturated solution of **7**‐**10** in dichloromethane. **7** crystallizes in the monoclinic space group *C*2/*c* with an anti‐assignment of the acetyl groups and shows π‐π stacking (d_π‐π_=3.34 Å). The molecules are stacked in a coplanar manner with alternating ketone and acetyl moiety. **8** crystallizes in the monoclinic space group *P*2_1_/*n* with a π‐π stacking distance of d_π‐π_=3.50 Å. In contrast to **7**, the methoxy functionalities are aligned to each other and four stacked molecule pillars are placed in a circular, twisted orientation. **9** with its bulkier TIPS‐groups crystallizes in the triclinic space group $P\bar{1}$ with a smaller π‐π stacking distance than in **7** and **8** of d_π‐π_=3.23 Å. The ketone functionality of the molecules is vertically aligned, and the *syn*‐oriented TIPS‐groups are in anti‐conformation and space filling with respect to the adjacent molecule (see Figure 1).

**Figure 2:**
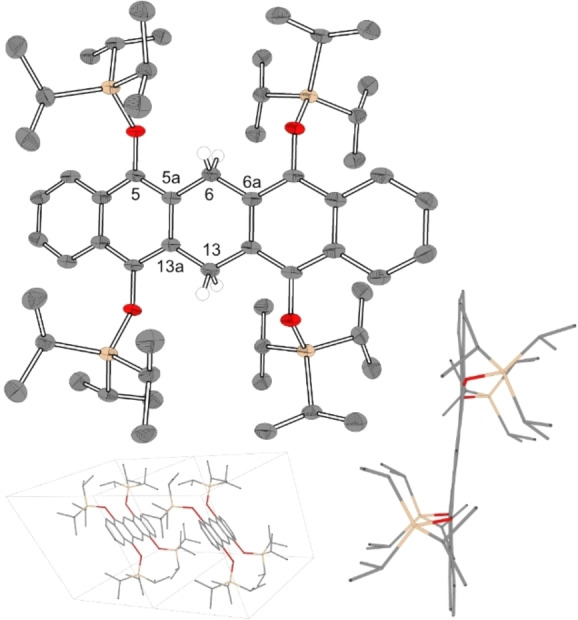
Solid state structures and packing of **10**. Most hydrogen atoms are omitted for clarity, 50 % probability. Selected Bond lengths C_5a_ – C_6_=1.5056(33) Å, C_6_ – C_6a_=1.5088(27) Å, C_5a_ – C_6_ – C_6a_=117.25(15)°.

Tetra‐substituted **10** crystallizes in the triclinic space group $P\bar{1}$ . Due to the spatial shielding of the bulky TIPS‐groups π‐stacking is essentially abolished, resulting in a closest interplanar distance of slipped π‐systems at a distance of 6.90 Å, which is displayed in Figure 2. From ^1^H‐NMR spectroscopy and HR‐mass spectrometry (FD^+^) data, it is confirmed that the central carbon atoms C_6_ and C_13_ are reduced to methylene groups. It would be expectable that these sp^3^ carbon atoms exhibit an out‐of‐plane arrangement. Single crystal diffraction data, however, have shown that the backbone is forced into a nearly planar conformation. Notably, electron density resembling two H atoms at positions identical to those of an ideally calculated CH_2_ group, which is found in the difference Fourier map below and above but not within the central ring plane at C_6_. If these two hydrogen atoms are added by calculation, the structure refinement showed a better R_1_ value for a sp^3^‐CH_2_ (R_1_=3.84) than for a sp^2^‐CH (R_1_=4.19) group.

In contrast to these experimental XRD results, gas phase calculations for parent unsubstituted 6,13‐dihydropentacene revealed, that the energy for the folded structure is 4 kcal/mol lower than for the planar structure.^[15]^ The DFT calculated structure of **10** (B3LYP/def2‐tzvpp) is also showing the minimum in a folded conformation. By varying the angle of the two naphthalene moieties in a relaxed potential energy surface scan (B3LYP/def2‐svp), a difference in energy of 3.3 kcal/mol was calculated between the planar and the boat conformation (*vide infra*). A rigid boat conformation would imply that the two methylene protons are chemically inequivalent, an AB spin system would be expected for *endo*‐ and *exo*‐protons. The observed singlet is expected to be the result of a rapid conformational inversion on the NMR time scale. However, variable temperature NMR studies on **10** in dichloromethane‐*d_2_* from room temperature to −80 °C (500 MHz) did not indicate any splitting of the singlet into doublets. Obviously, the inversion barrier is too low to detect the minimum structure of **10** as the only species in a low‐temperature scenario by means of ^1^H NMR spectroscopy. With reference to other experimentally documented examples of planar dihydropentacenes[^15^, ^36^] we assume, that the fully planar solid state molecular structure of **10** is induced by packing effects of the bulky substituents and to a minor part by intermolecular π‐stacking. Characteristic bond distances and angles of literature known and structurally characterized dihydropentacene derivatives are compared in the SI (Table S3). The bond distances of the central methylene moiety of **10** (C_5a_ – C_6_=1.5056 Å, C_6_ – C_6a_=1.5088 Å) are close to the bond distance of the 6,13‐dihydropentacene (C_5a_ – C_6_=1.475 Å to C_6_ – C_6a_=1.524 Å).^[14]^ These bonds are significantly longer compared to pentacene (C_5a_ – C_6_=1.4336 Å, C_6_ – C_6a_=1.4345 Å).^[14]^ The bond distance of **10** (C_5a_ – C_13a_=1.4174 Å) is smaller than those of parent pentacene (C_5a_ – C_13a_=1.4417 Å) and is more comparable to those of 6,13‐dihydropentacene (C_5a_ – C_13a_=1.4251 Å).^[14]^ In addition to the bond lengths, the internal bond angle α(C_5a_ – C_6_ –C_6a_)=117.25° of the methylene carbon atom for **10** is smaller when compared to planar pentacene α(C_5a_ – C_6_ –C_6a_)=118°. 6,13‐Dihydropentacene exhibits a bent structure which yields to a sharper bond angle of α(C_5a_ – C_6_ –C_6a_)=113.7°.^[14]^ These results are comparable to other published solid state structures of 6,13‐dihydropentacenes and their derivatives, which show also a planar conformation of the central backbone.[^15^, ^36^] Their methylene C−C bond distances ranged from 1.5272 Å to 1.5406 Å, their bond angles C_5a_ – C_6_ –C_6a_ ranged from 114.3° to 117.3°.

### Optoelectronic Properties

**Figure 3:**
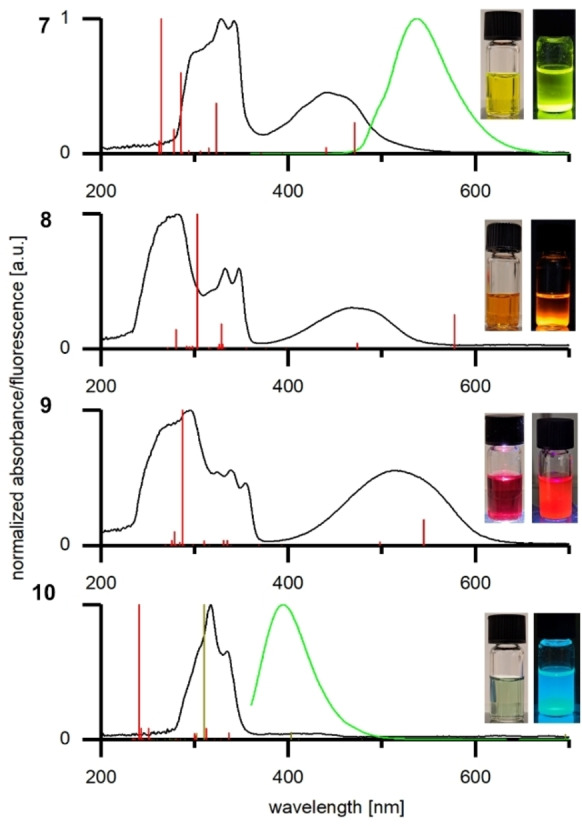
UV/‐Vis (black lines, dichloromethane c ≈10^−5^ M) and photoluminescence (green lines, λ_Exp_=350 nm, dichloromethane c ≈10^−7^ M) spectra recorded of **7‐10**. Calculated solvent dependent TD‐DFT absorption spectra (B3LYP/def2‐TZVPP level of theory, dichloromethane) in red lines and tetra(OTPS)pentacene (**10**‐2H, golden lines). The inserted pictures display solutions of corresponding molecules in dichloromethane at ambient light (left) and under UV irradiation (right).

**Figure 4:**
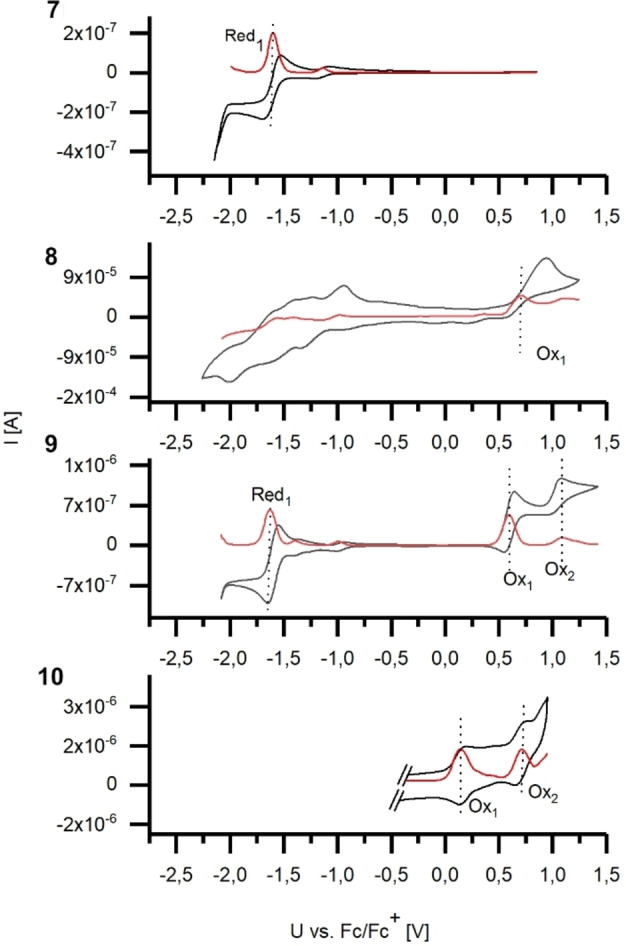
Cyclic voltammograms (measured in anhydrous dichloromethane under inert atmosphere, 0.1 M Bu_4_NPF_6_, 50 mVs^−1^, glassy carbon electrode) and their corresponding differential pulse voltammograms (DPV, red lines, 10 mV s^−1^). Ferrocene addition for means of internal calibration.

**Figure 5:**
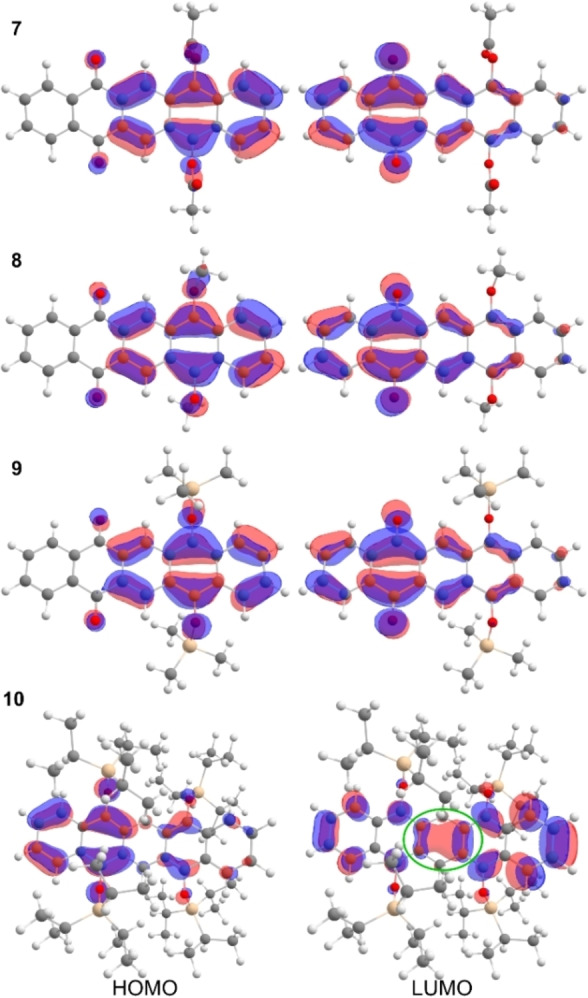
DFT‐calculated Kohn‐Sham frontier molecular orbitals of **7**, **8**, **9**, **10** (B3LYP/def2‐TZVPP level of theory, isopropyl groups are simplified as methyl groups for **9**), iso‐value: 0.03). Homoconjugation in **10** is pointed out by the green circle.

The following section correlates experimental UV/Vis and CV data with results from DFT calculations. In order to study the influence of different substituents on the optical properties, UV/Vis and qualitative photoluminescence spectra of pentacene derivatives **7‐10** have been measured in dichloromethane (Figure 3). The absorptions in the UV region are more structured, the lower energy bands in the visible region are broader and of lower intensity. This indicates weak electronic transitions resulting from the rigid π‐backbone.^[47]^ Acetate **7** shows absorption maxima at 328 nm and 441 nm in the UV/Vis range, respectively, which is identical to the literature reported (330 nm and 450 nm) for **7**.^[40]^ The stronger electron‐donating effect of ‐OMe compared to ‐OAc substituents results in a bathochromic shift of the absorption maximum from 441 nm (**7**) to 468 nm (**8**). Chromophore **9** comprising ‐OTIPS substituents displays the most red‐shifted absorption maximum at 515 nm. A similar trend has been observed in a series of ‐OR substituted violanthrenes.^[30]^ In sharp contrast, dihydropentacene **10** displays an extremely broad absorption of low intensity in the visible light region, resulting in a pale green solution when dissolved in dichloromethane (Figure 3). The pronounced signal in the UV region of **10** indicates the presence of two naphthalene subunits. Very similar absorptions (275 nm to 350 nm) are observed in parent 1,4‐bis(OTIPS) naphthalene.^[48]^ In contrast, pentacenes such as **5** show a broad absorption in the visible range from 400 to 750 nm.^[49]^ We suggest to attribute the broad signal between 360 nm and 460 nm in **10** to a certain contribution of homoconjugation between the two naphthalene moieties.[^50^, ^51^] In order to gain further insight into the energy transitions, TD‐DFT calculations at the B3LYP/def2‐TZVPP level of theory were performed. UV/Vis transition wavelengths of the molecules in their optimized geometries are shown in Figure 3 and support the experimental observations for compounds **7** to **9** in the UV range. The visible band is also well represented, and the transition wavelengths increase corresponding to the experimental spectra. According to our TD‐DFT analyses, the lowest energy transitions originate from HOMO‐LUMO (S0←S1) transitions. For **10**, no transition in the visible range was found from calculations. Furthermore, the transition energy with the highest contribution shows a significant shift to higher energies when compared to the experimental data. Therefore, an optimized geometry of tetra(OTIPS)pentacene was calculated which displays a calculated transition energy in the visible range (see Figure 3, **10**, golden line, λ_max_ (vis)= 410 nm). This absorption matches perfectly with the experimental absorption of **10** taken from UV/Vis spectroscopy, λ_max_ (vis)= 406 nm. This broad absorption between 360 nm and 460 nm might be explained by homoconjugation of the two naphthalene subunits. Support for this interpretation comes from DFT‐calculated Kohn‐Sham frontier molecular orbitals of **10** displayed in Figure 4 and discussed below. The optimized structure of **10** displays an homoconjugation between the two naphthalene moieties in the HOMO‐2, LUMO and LUMO+2, which can be seen in Figure 5 and Figure S21, SI.

Only **7** and **10** show a photoluminescence signal at a maximum of 537 nm and 395 nm, respectively, visualized by the green lines in Figure 3. In general, fluorescence intensities of **7** and **10** are low, resulting in non‐quantifiable quantum yields. In addition, **8** and **9** did not show any observable fluorescence signals. That leads to the conclusion that non‐radiative decay pathways of electronic excitation are favored over photoluminescence in the case of **7–10**.

The electrochemical properties of molecules **7**‐**10** were evaluated by means of cyclic voltammetry (CV) in dichloromethane/0.1 M Bu_4_NPF_6_ and their redox half wave potentials were determined with respect ferrocene/ferrocenium Fc/Fc^+^ redox couple (E_vac_=4.8 eV)^[52]^ added directly after recorded cycle and in combination with differential pulse voltammetry (DPV). The graphs are displayed in Figure 5. Electron poor **7** shows a reversible redox event at −1.60 V, but no oxidation event in the electrochemical window of dichloromethane has been observed. The methoxy‐substituted compound **8** shows several irreversible oxidation and reduction events. Under oxidizing conditions, the cyclic voltammogram reveals an irreversible oxidation at 0.71 V. During the measurements, a black precipitate was formed which indicates the electrochemical instability of **8**. The more electron‐rich compound **9** shows a reversible redox wave at −1.62 V and two partially reversible redox events at 0.60 V and 1.09 V, respectively. The over‐reduced molecule **10**, as a highly electron rich compound, shows two partially reversible redox events at 0.15 V and 0.70 V but no redox event under reducing conditions could be detected in the electrochemical window of dichloromethane. The reduction events accessible for **7** (−1.60 V and −1.12 V [very small]) and **9** (−1.62 V und −1.00 V [very small]) are very similar in terms of their potential and reversibility. They are most likely resulting from the quinone moiety. The CV of parent anthraquinone reference, recorded under the same conditions, gave reduction potentials at −0.81 V and −1.65 V. In contrast to **9**, the oxidation of **7** was not possible under the conditions of the cyclic voltammetry experiment due to the electron withdrawing acetyl residues. In contrast to **7** the highly electron rich compound **10** does not show a reduction process but two oxidation half waves, which are shifted to lower voltage levels then for **9**.

### DFT Calculations

**Figure 6:**
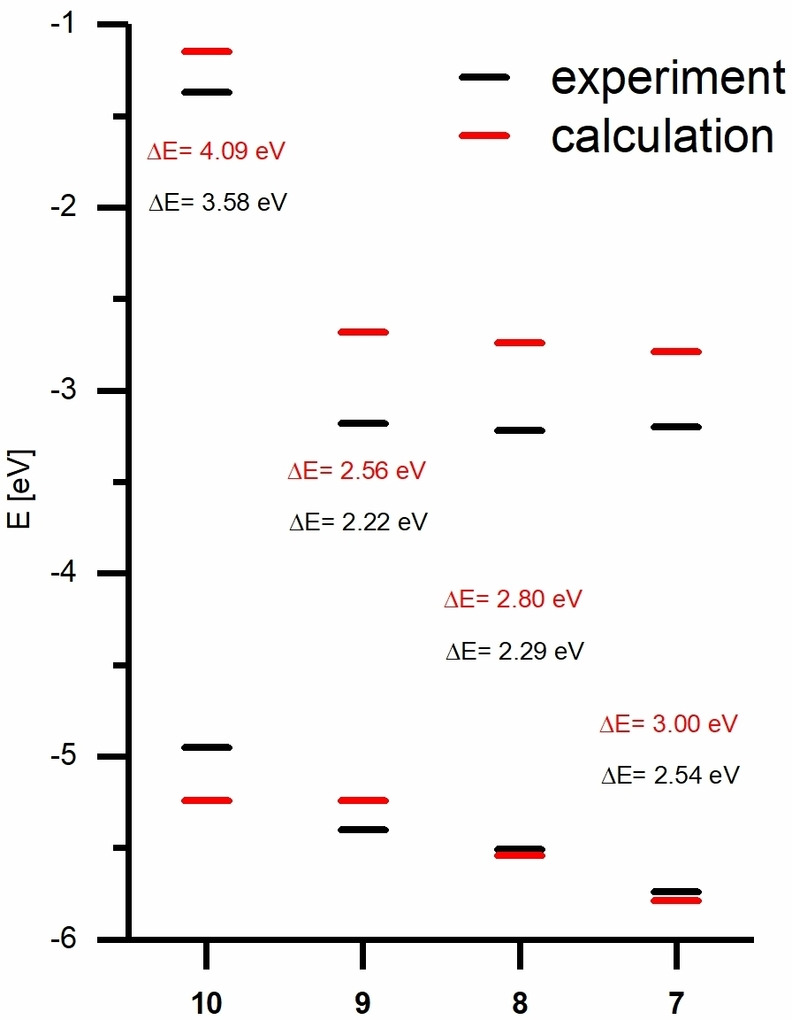
Energy diagram of experimental and calculated HOMO and LUMO energies of compounds **7–10**. Experimental energies were calculated as described in Table 1. Calculated energies were determined from B3LYP/def2‐TZVPP level of theory calculations.

In order to theoretically evaluate the electronic structure of **7–10**, quantum chemical calculations were performed. A benchmark study had been carried out to find the best computational model for these compounds and acetate **7** was chosen as a test system. Different functionals (B3LYP, PBE, M06‐2X, wB97X) and basis sets (def2‐TZVPP, def2‐TZVPPD, 6–311G*) were used with and without dispersion corrections. The influence of dichloromethane as solvent was investigated using the Conductor‐like Polarizable Continuum Model (CPCM). In general, adding dispersion correction gave the same HOMO/LUMO energies, but led to optimized geometries without an imaginary vibrational mode. Inclusion of the solvent resulted in a small increase of the HOMO/LUMO energies, which makes the calculation more accurate. The results can be found in the SI in Figure S6. B3LYP/def2‐TZVPP including dichloromethane has led to the best results compared to experimental values and was therefore used as the default level of theory. As expected, the HOMO of **7**‐**9** is delocalized over the anthrahydroquinone subunit, while the LUMO is delocalized over the anthraquinone backbone. The HOMO energy increases with increasing donor capability of the O‐substituents yielding energies ranging from −5.79 eV (**7**) to −5.24 eV (**9**). The calculated LUMO energies for molecules **7–9** have similar values in the range of −2.68 to −2.79 eV attributed to the quinoid moiety. The HOMO/LUMO energy gaps are in a range of 3.00 eV (**7**) and 2.56 eV (**9**). Experimantal and calculated values are summarized in Figure 6.

Contrastingly, in compound **10**, the π‐electron systems of both naphthalene‐type subunits strongly contribute to both the HOMO and the LUMO. Compared to the XRD solid‐state structure, the optimized gas phase structure displays a boat conformation at the central ring with an angle of 139° between the two naphthalene moieties. Calculations varying this angle between 130° and 200° in a relaxed potential energy surface scan (B3LYP/def2‐svp with Grimme D3 dispersion corrections) revealed that the local minimum is at an angle of ca. 143°. The maximum is found at an angle of 180°, which is only 3.3°kcal/mol higher in energy in this computational model. This indicates a low barrier for the bending of compound **10** about the axis formally connecting the two CH_2_ carbon atoms of the central six‐membered ring. This finding supports packing effects to account for the stabilization of the planar conformer of compound **10** in the solid state. Despite the unsaturated central six‐membered ring, the LUMO of **10** displays considerable delocalization between the two naphthalene π‐systems, which makes homoconjugation plausible (Figure 5, green circle). Additionally, homoconjugation can be observed in the HOMO‐2 and in LUMO+2 (Figure S23). The HOMO energy for **10** is −5.24 eV and the LUMO energy of the electron rich compound is raised to −1.09 eV. The calculated HOMO/LUMO energy gap of **10** is 4.09 eV, which is comparable to the frontier orbital energy gap of naphthalene of 4.78 eV (HOMO=−6.12 eV, LUMO=−1.34 eV^[53]^). The experimental and calculated optoelectronical parameters of **7–10** are summarized in Table 1, which are determined by literature known methods.[^52^, ^54^] The experimental HOMO energies are in good agreement with the calculated data, however the LUMO energies differ by 0.31 eV to 0.50 eV from the experimental data, which results in a maximal difference of the HOMO/LUMO energy gap between calculated and experimental data of 0.51 eV. In general, the B3LYP/def2‐TZVPP level of theory suits to the molecules **7–10** and their experimental properties and represents the trend of the energies. The value of all LUMO energies however is calculated slightly too high, but nevertheless the trend is preserved.

**Table 1:** Experimental and calculated optoelectronical parameters of **7–10**.

Compound	UV/‐Vis/PL spectra		Cyclic voltammetry					DFT (B3LYP/def2‐TZVPP)		
	λ_max, abs._ [nm]	λ_max, em._ [nm]	E_1/2, Red1/2._ [V]	E_1/2, Ox1/2._ [V]	E_HOMO, exp._ [eV]	E_LUMO, exp_. [eV]	E_gap, exp._ [eV]	E_HOMO, DFT_ [eV]	E_LUMO, DFT_ [eV]	E_gap, DFT_ [eV]
**7**	328/441	537	−1.60	‐	−5.74 ^[c]^	−3.20^[a]^	2.54^[d]^ [488]	−5.79	−2.79	3.00
**8**	283/468	–	–	0.71	−5.51^[b]^	−3.22^[c]^	2.29^[e]^ [538]	−5.54	−2.74	2.80
**9**	294/515	–	−1.62	0.60	−5.40^[b]^	−3.18^[a]^	2.22^[c]^	−5.24	−2.68	2.56
**10**	318/406	395	–	0.15	−4.95^[b]^	−1.37^[c]^	3.58^[d]^ [346]	−5.24	−1.15	4.09

[a] E_LUMO_=−4.8 eV^[52]^−E_1/2,Red1_, [b] E_HOMO_=−4.8 eV^[52]^−E_1/2,Ox1_, [c] E_HOMO_=E_LUMO_−E_gap, opt_, [d] determined from intersection wavelength of normalized absorption and emission spectra, [e] determined from the optical edge^[54]^

## Conclusions

In summary, we reported the synthesis of partially oxidized and reduced pentacene derivatives starting from commercially available and easily accessible 5,7,12,14‐pentacenetetrone, metallic potassium or zinc and alkyl, acetyl and silyl electrophiles. The partial reduction of one quinone moiety led to substituted anthracene derivatives **7–9**. Further reduction of the other quinone moiety did not lead to substituted pentacenes but to an over‐reduced dihydropentacene **10**. This indicates that the strategy of reductive aromatization and functionalization tends to yield more stable partially oxidized and reduced pentacene derivatives containing more than one Clar sextet as present in pentacene. Optical and electronic properties of the compounds **7–10** were evaluated by UV/Vis, by qualitative fluorescence spectroscopy, and by cyclovoltammetry. According to cyclic voltammetry the HOMO energy level of the substituted acenes could be tuned between −5.74 eV (**7**) and −4.95 eV (**10**) while the LUMO located at the remaining quinone (**7**‐**9**) is observed at around −3.20 eV and −1.37 eV for **10**, respectively. Corresponding UV/Vis spectroscopy showed a bathochromic shift from 441 nm (**7**) for electron donating ‐OTIPS to 515 nm (**9**) for less electron donating ‐OAc substituents. These optoelectronic properties were supported by (TD)‐DFT calculations that follow the trend of experimental data. Single crystal X‐ray analyses of **7–10** gave insight in their spatial arrangement and π‐π‐stacking distances. The planar central ring of 6,13‐dihydropentacene **10** in the solid‐state lattice structure is the most notable feature. Such planar dihydropentacene rings have been observed in two other literature known examples. The relaxed gas phase structure of **10** displays a bent conformation. This structural diversity could be the focus of a more detailed theoretical investigation.

## Experimental Section

**7**: 5,7,12,14‐Pentacenetetrone (100 mg, 0.30 mmol, 1.00 eq.) was charged into a Schlenk tube. Inside the glovebox, potassium (46.2 mg, 1.18 mmol, 4.00 eq.) was added. Dry 1,4‐dioxane (20.0 ml) was added to yield a brown suspension and the mixture was heated to 90 °C for 16 h. The purple solution was cooled to room temperature and acetyl chloride (0.20 ml, 2.36 mmol, 8.00 eq.) was added. The solution was stirred at room temperature for 2 h. The yellow solution was filtered through a filter paper and was washed with dichloromethane. The solvent was removed *in vacuo*. It was recrystallized from dichloromethane and pentane to yield **7** (84.0 mg, 0.20 mmol, 67 %) as yellow needles.

^1^H NMR (300 MHz, CDCl_3_) δ (ppm): 9.03 (s, 2H, H_arom_), 8.42 (dd, ^3^J=5.8, 3.4 Hz, 2H, H_arom_), 8.02 (dd, ^3^J=6.7, 3.2 Hz, 2H, H_arom_), 7.86 (dd, ^3^J=5.9, 3.2 Hz, 2H, H_arom_), 7.65 (dd, ^3^J=6.8, 3.1 Hz, 2H, H_arom_), 2.74 (s, 6H, CH_3_).

^13^C{1H} NMR (75 MHz, CDCl_3_): δ (ppm): 182.30, 169.28, 143.25, 134.72, 134.43, 129.33, 128.39, 127.71, 126.66, 125.09, 124.43, 122.42, 20.87.

HR‐MS (FD^+^) *m/z* calcd. for [M]^+^ C_26_H_16_O_6_: 424.09469; found: 424.09461.

IR (ATR): $\tilde{\nu }=$ 3070(vw), 2960 (vw), 2930 (vw), 1771 (m), 1752 (m), 1674 (m),1590 (m), 1447 (m), 1423 (m), 1360 (m), 1323 (s), 1273 (m), 1182 (s), 1045 (s), 975 (m), 888 (m), 798 (m), 768 (m), 745 (m), 708 (s), 568 (m).

**8**: 5,7,12,14‐Pentacenetetrone (100 mg, 0.30 mmol, 1.00 eq.) was charged into a Schlenk tube. Inside the glovebox, potassium (69.3 mg, 1.77 mmol, 6.00 eq.) was added. Dry 1,4‐dioxane (20.0 ml) was added to yield a brown suspension and the mixture was heated to 90 °C for 5 h. The purple solution was cooled to room temperature, methyl iodide (0.20 ml, 3.25 mmol, 11.0 eq.) it was stirred at room temperature overnight. The green suspension was filtered using a funnel and filter paper and was washed with dichloromethane to yield an orange solution. The solvent was removed *in vacuo* to obtain an orange solid, which was recrystallized from dichloromethane and pentane to yield **8** (9.00 mg, 0.02 mmol, 8 %) as orange needles.

^1^H NMR (300 MHz, CDCl_3_) δ (ppm): 9.36 (s, 2H, H_arom_), 8.44 (dd, ^3^J=5.8, 3.3 Hz, 2H, H_arom_), 8.36 (dd, ^3^J=6.7, 3.2 Hz, 2H, H_arom_), 7.84 (dd, ^3^J=5.9, 3.3 Hz, 2H, H_arom_), 7.62 (dd, ^3^J=6.7, 3.2 Hz, 2H, H_arom_), 4.23 (s, 6H, CH_3_).

^13^C{1H} NMR could not be recorded due to low solubility.

HR‐MS (APCI) *m/z* calcd. for [M+H]^+^ C_24_H_16_O_4_: 369.1121; found: 369.1120.

**9**: 5,7,12,14‐Pentacenetetrone (100 mg, 0.30 mmol, 1.00 eq.) and Zn powder (155 mg, 2.36 mmol, 8.00 eq.) were dissolved in dry 1,4‐dioxane (20.0 ml) to yield a brownish suspension. TIPS‐Cl (0.42 ml, 2.36 mmol, 8.00 eq.) and imidazole (160 mg, 2.36 mmol, 8.00 eq.) were added and the purple solution was stirred at 90 °C for 3 d. The mixture was filtered through a pad of neutral alumina and washed with dichloromethane. The solvent was removed under reduced pressure and the resulting dark red solid was washed with methanol (2 x 2 ml) to obtain **9** (147 mg, 0.23 mmol, 76 %) as red solid.

^1^H NMR (300 MHz, CDCl_3_): δ (ppm): 9.35 (s, 2H, H_arom_), 8.44 (dd, ^3^J=5.8, 3.3 Hz, 2H, H_arom_), 8.35 (dd, ^3^J=6.7, 3.3 Hz, 2H, H_arom_), 7.81 (dd, ^3^J=5.9, 3.3 Hz, 2H, H_arom_), 7.56 (dd, ^3^J=6.8, 3.2 Hz, 2H, H_arom_), 1.55 (p, ^3^J=7.5 Hz, 6H, Si‐CHR_2_), 1.15 (d, ^3^J=7.5 Hz, 36H, CH_3_).

^13^C{^1^H} NMR (75 MHz, CDCl_3_): δ (ppm): 182.83, 146.56, 135.34, 134.02, 127.63, 127.37, 126.76, 126.61, 123.76, 123.12, 18.20, 14.28.

HR‐MS (FD^+^) *m/z* calcd. for [M]^+^ C_40_H_52_O_4_Si_2_: 652.34041; found: 652.34230.

IR (ATR): $\tilde{\nu }=$ 2944 (m), 2889 (w), 2865 (m), 1669 (m), 1656 (w), 1579 (m), 1459 (m), 1441 (m), 1382 (m), 1324 (m), 1270 (s), 1251 (s), 1146 (m), 1062 (m), 971 (m), 876 (s), 785 (m), 756 (m), 672 (vs), 501 (m).

**10**: 5,7,12,14‐Pentacenetetrone (100 mg, 0.30 mmol, 1.00 eq.) was charged into a Schlenk tube. Inside a glovebox, potassium (70 mg, 1.77 mmol, 6.00 eq.) was added. Dry 1,4‐dioxane (20.0 ml) was added to yield a brown suspension and the mixture was heated to 90 °C for 5 h. The purple solution was cooled to room temperature and TIPS‐Cl (0.63 ml, 2.36 mmol, 10.0 eq.) was added. The solution was stirred at 90 °C for 16 h. It was filtered under inert atmosphere using a glass frit yielding a dark brown solution. The solvent was evaporated, and dry methanol was added (10 ml). A green precipitate was formed, the mixture was stirred for 1 h at room temperature and the solvent was removed. The green precipitate was filtered through a pad of celite and washed with dry *n*‐pentane (20 ml). The filtrate was discarded. Dry dichloromethane (100 ml) was passed through the celite pad, the product dissolved and the filtrate was collected. The solvent was removed under reduced pressure to yield **10** (148 mg, 0.15 mmol, 52 %) as a green solid.

^1^H NMR (300 MHz, CD_2_Cl_2_): δ (ppm): 8.10 (dd, *J*=6.4, 3.3 Hz, 4H, H_arom._), 7.32 (dd, ^3^ *J*=6.5, 3.2 Hz, 4H, H_arom._), 4.27 (s, 4H, H_benz._), 1.44 (p, ^3^ *J*=7.5 Hz, 12H, H_alk_), 1.06 (d, ^3^ *J*=7.3 Hz, 72H, H_alk_).

^13^C{^1^H} NMR (75 MHz, CD_2_Cl_2_): δ (ppm): 143.22, 127.47, 124.04, 123.90, 122.43, 26.13, 18.45, 14.60.

^29^Si{^1^H} NMR (60 MHz, CD_2_Cl_2_): δ (ppm): 15.68.

HR‐MS (FD^+^) *m/z* calcd. for [M]^+^ C_58_H_96_O_4_Si_4_: 968.63856; found: 968.64174

IR (ATR): $\tilde{\nu }=$ 2943 (m), 2865 (m), 1463 (m), 1369 (s), 1257 (m), 1181 (m), 1061 (s), 1012 (m), 881 (m), 825 (m), 796 (m), 761 (m), 670 (s), 519 (m), 494 (m).

## Conflict of Interests

The authors declare no conflict of interest.

1

## Supporting information

As a service to our authors and readers, this journal provides supporting information supplied by the authors. Such materials are peer reviewed and may be re‐organized for online delivery, but are not copy‐edited or typeset. Technical support issues arising from supporting information (other than missing files) should be addressed to the authors.

Supporting Information

## Data Availability

The data that support the findings of this study are available from the corresponding author upon reasonable request.
