# Supplementary material for: Reductive Aromatization of 5,7,12,14‐Pentacenetetrone: Approach Towards Substituted Pentacenes?
Source: Chemistry. 2024 Dec 30;31(8):e202403929. doi: 10.1002/chem.202403929 (PMC11803357; doi:10.1002/chem.202403929)
Supplement: Supplementary file 1 — Supporting Information [file CHEM-31-e202403929-s001.pdf]

Chemistry–A European Journal

Supporting Information

Reductive Aromatization of 5,7,12,14-Pentacenetetrone: Approach Towards Substituted Pentacenes?

Olaf A. Kleykamp, Eugen Sharikow, Andreas Stoy, Xiulan Xie, Crispin Lichtenberg, and Jörg Sundermeyer*

Reductive Aromatization of 5,7,12,14-Pentacenetetrone: Approach Towards Substituted Pentacenes?

Electronic Supplementary Information

Content

Materials, Methods and Devices S1
Single crystal S2
NMR Spectra S15
Mass Spectra S23
DFT and TD-DFT results S26

Materials, Methods and Devices

All reactions were carried out under inert atmosphere using standard SCHLENK techniques. Air and/or moisture sensitive substances were stored in a nitrogen flushed glovebox. Solvents were purified according to common literature procedures and stored under an inert atmosphere over molecular sieves (3 Å or 4 Å).[1] 1H, 13C NMR spectra were recorded on a Bruker Avance III HD 250, Avance II 300, Avance III HD 300 or Avance III HD 500 spectrometer. NMR-Chemical shift δ is denoted relatively to $SiMe_4$ for ^1H, ^{13}C. ^1H and ^{13}C NMR spectra were referenced to the solvent signals.[2] Multiplicity is abbreviated as follows: s (singlet), d (doublet), t (triplet), q (quartet), m (multiplet), br (broad signal). HR-APCI mass spectra were acquired with a LTQ-FT Ultra mass spectrometer (Thermo Fischer Scientific). The resolution was set to 100.000. HR-FD mass spectra were acquired with a AccuTOF GCv 4G (JEOL) Time of Flight (TOF) mass spectrometer. An internal or external standard was used for drift time correction. The LIFDI ion source and FD-emitters were purchased from Linden ChroMasSpec GmbH (Bremen, Germany). Absorption spectra were recorded with an Avance AvaSpect-2048 UV/Vis/NIR spectrophotometer in 10 mm cuvettes in dichloromethane or toluene with concentrations of 10 *µ*M with a scan rate of 600 nm/min. Emission spectra were recorded with a JASCO FP6000 fluorescence spectrophotometer in 10 mm cuvettes in dichloromethane with a scan rate of 600 nm/min. Cyclic voltammetry (CV) and differential pulse voltammetry (DPV) measurements were carried out on a RHD instruments TSC 1600 closed electrochemical workstation (working electrode: glassy carbon; counter electrode: platinum crucible; reference electrode: platinum wire (pseudo reference electrode under nitrogen atmosphere in a glovebox (Labmaster 130, mBraun). The samples were measured in dichloromethane and calibrated using ferrocene as internal standard after measurements. Dichloromethane was filtered through an aluminum oxide pad prior to use. Tetrabutylammonium hexafluorophosphate (Bu_4NPF_6; >99.0 %) was used as the electrolyte for electrochemical analysis. The measurements were carried out at a concentration of 100 mmol/L of electrolyte. IR spectra are recorded with a Bruker Alpha FT-IR spectrometer with Platinum ATR sampling.

The data collection for the single-crystal structure determination was performed on a Stoe Stadivari diffractometer or a Bruker D8 Quest diffractometer by the X-ray service of Fachbereich Chemie, Universität Marburg. Information concerning the used hardware, and software used for data collection, cell refinement and data reduction as well as structure refinement can be reviewed in the electronic supplement tables and CCDC 2257477 – 2257479 – 2257478 – 2257475. After solution (SHELXT)[3] and refinement process (SHELXL 2017/1)[4] the data were validated by using Platon.[5] All graphic representations were created with Diamond 4. Deposition numbers CCDC 2257477 – 2257479 – 2257478 – 2257475 contain the supplementary crystallographic data for this paper. These data are provided free of charge by the joint Cambridge Crystallographic Data Centre and Fachinformationszentrum Karlsruhe via www.ccdc.cam.ac.uk/data_request/cif.

All calculations were performed at the B3LYP[6–9]/def2-tzvpp[10] level of theory with the Conductor-like Polarizable Continuum Model (CPCM).[11] All structures were optimized without geometrical constraints and their vibrational frequencies were computed in order to confirm the energy surface of the optimized geometries as a global minimum. All calculations have been carried out using the ORCA program package.[12] Atom pairwise dispersion correction by Grimme *et al* . as well as auxiliary basis sets by Weigend *et al*.[10,13] were utilized. The vibrational entropy was computed according to Grimme.[14] Rotational entropy computed according to Herzberg.[15]

The EPR spectra were recorded on a Bruker Magnettech ESR5000 spectrometer operating in the X-Band (9.4 GHz). All samples were prepared in an argon-filled glovebox and transferred into a quartz glass tube prior to data collection. All measurements were done under an atmosphere of purified argon.

Single Crystal X-ray Diffraction Analyses

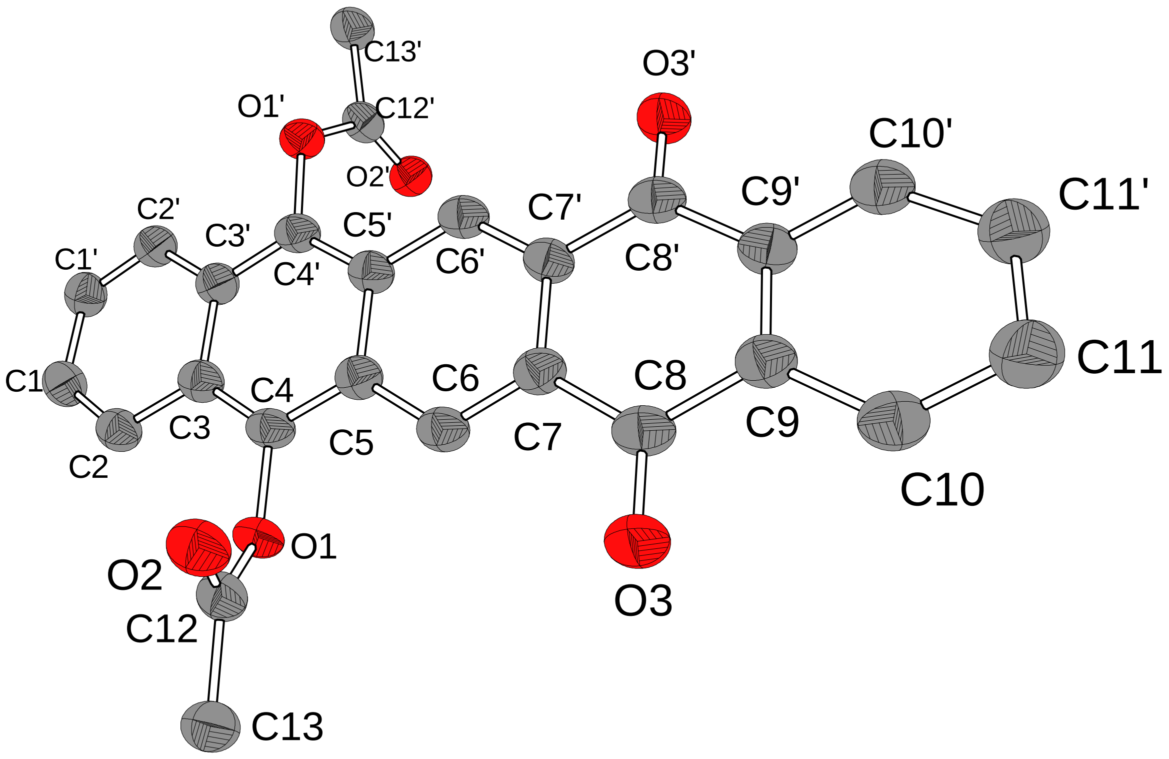

Figure S1: Solid state structures and packing of **7**. Hydrogen atoms are omitted for clarity and thermal ellipsoids are shown at 50% probability level. Symmetry transformations: 1 -x+1,y,-z+1/2.

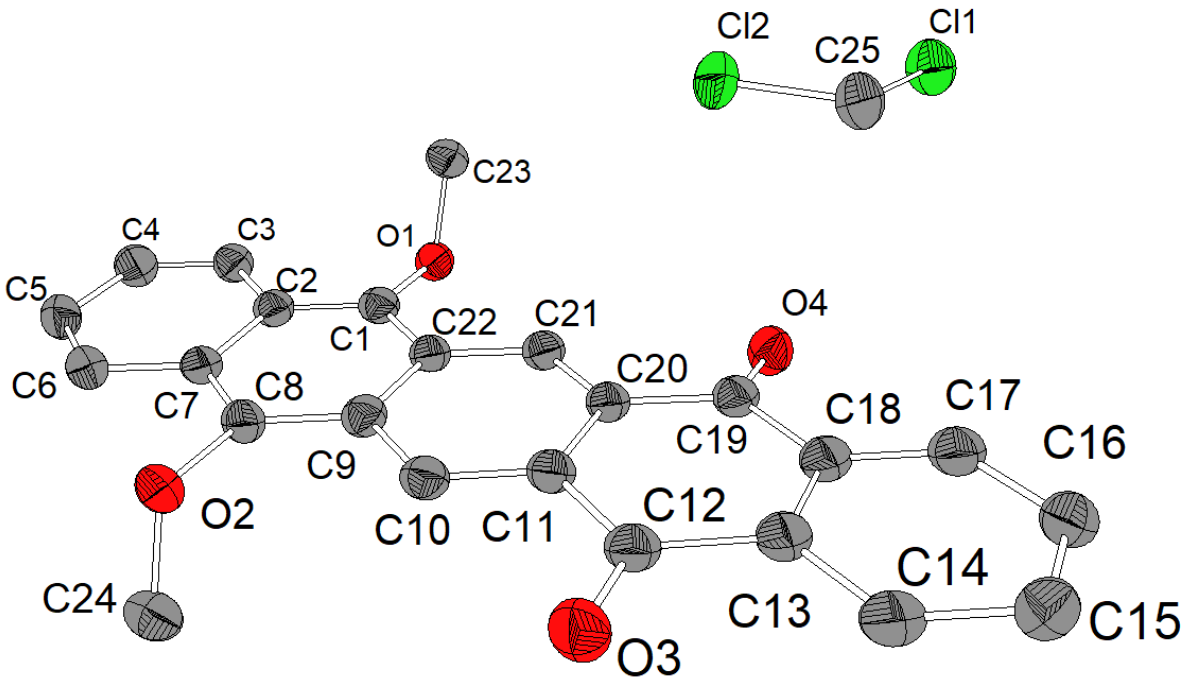

Figure S2: Solid state structures and packing of **8**. Hydrogen atoms are omitted for clarity and thermal ellipsoids are shown at 50% probability level.

Table S1: Single crystal X-ray diffraction data for **7** and **8**.

Identification code	**7**	**8**
CCDC code	2257479	2257478
Empirical formula	C26 H16 O6	C25 H18 Cl2 O4
Formula weight	424.39	453.29
Temperature	100(2) K	100(2) K
Wavelength	1.54178 Å	1.54178 Å
Crystal system	Monoclinic	Monoclinic
Space group	C 2/c	P 21/n
Unit cell dimensions	a = 11.1993(5) Å	a = 4.07190(10) Å
	b = 22.7657(14) Å	b = 19.2881(5) Å
	c = 7.3856(3) Å	c = 25.3287(8) Å
	α = 90°	α= 90°
	β= 95.877(4)°	β= 91.381(3)°
	γ = 90°	γ = 90°
Volume	1873.13(16) $Å^3$	1988.72(9) $Å^3$
Z	4	4
Density (calculated)	1.505 Mg/m^3	1.514 Mg/m^3
Absorption coefficient	0.891 mm^{-1}	3.209 mm^{-1}
F(000)	880	936
Crystal size	0.143 x 0.104 x 0.061 mm^3	0.276 x 0.073 x 0.055 mm^3
Theta range for data collection	3.883 to 75.804°.	2.880 to 76.030°.
Index ranges	-14<=h<=13, -26<=k<=28, -4<=l<=9	-4<=h<=5, -23<=k<=14, -31<=l<=31
Reflections collected	12173	26353
Independent reflections	1916 [R(int) = 0.0745]	4105 [R(int) = 0.0742]
Completeness to theta = 67.679°	99.7 %	99.6 %
Absorption correction	Semi-empirical from equivalents	Semi-empirical from equivalents
Max. and min. transmission	1.0000 and 0.1566	1.0000 and 0.2071
Refinement method	Full-matrix least-squares on F^2	Full-matrix least-squares on F^2
Data / restraints / parameters	1916 / 0 / 147	4105 / 0 / 282
Goodness-of-fit on F^2	0.965	0.984
Final R indices [I>2sigma(I)]	R1 = 0.0619, wR2 = 0.1606	R1 = 0.0538, wR2 = 0.1506
R indices (all data)	R1 = 0.0903, wR2 = 0.1813	R1 = 0.0706, wR2 = 0.1572
Extinction coefficient	0.0013(3)	n/a
Largest diff. peak and hole	0.335 and -0.266 $e.Å^{-3}$	0.348 and -0.503 $e.Å^{-3}$

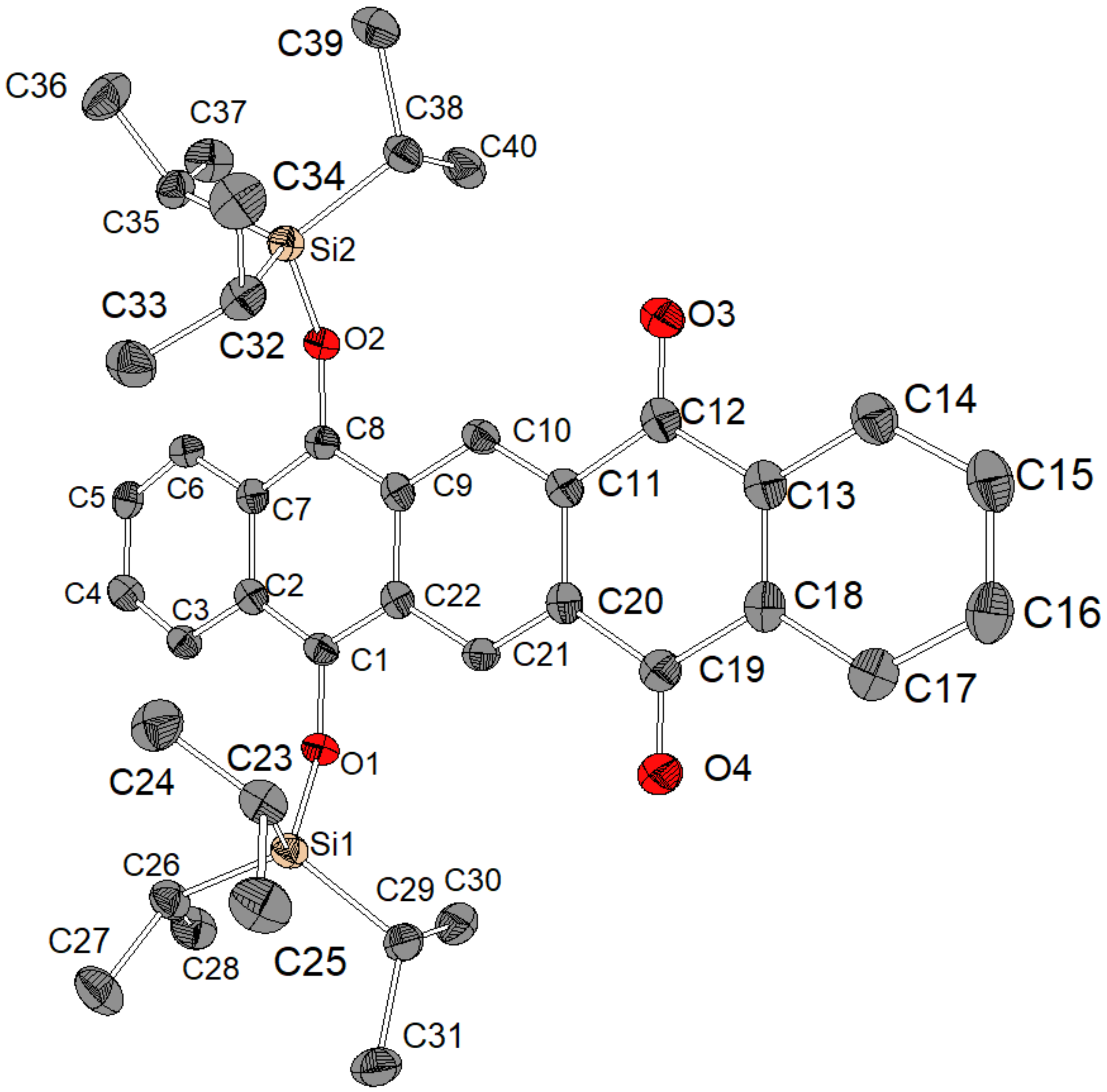

Figure S3: Solid state structures and packing of **9**. Hydrogen atoms are omitted for clarity and thermal ellipsoids are shown at 50% probability level.

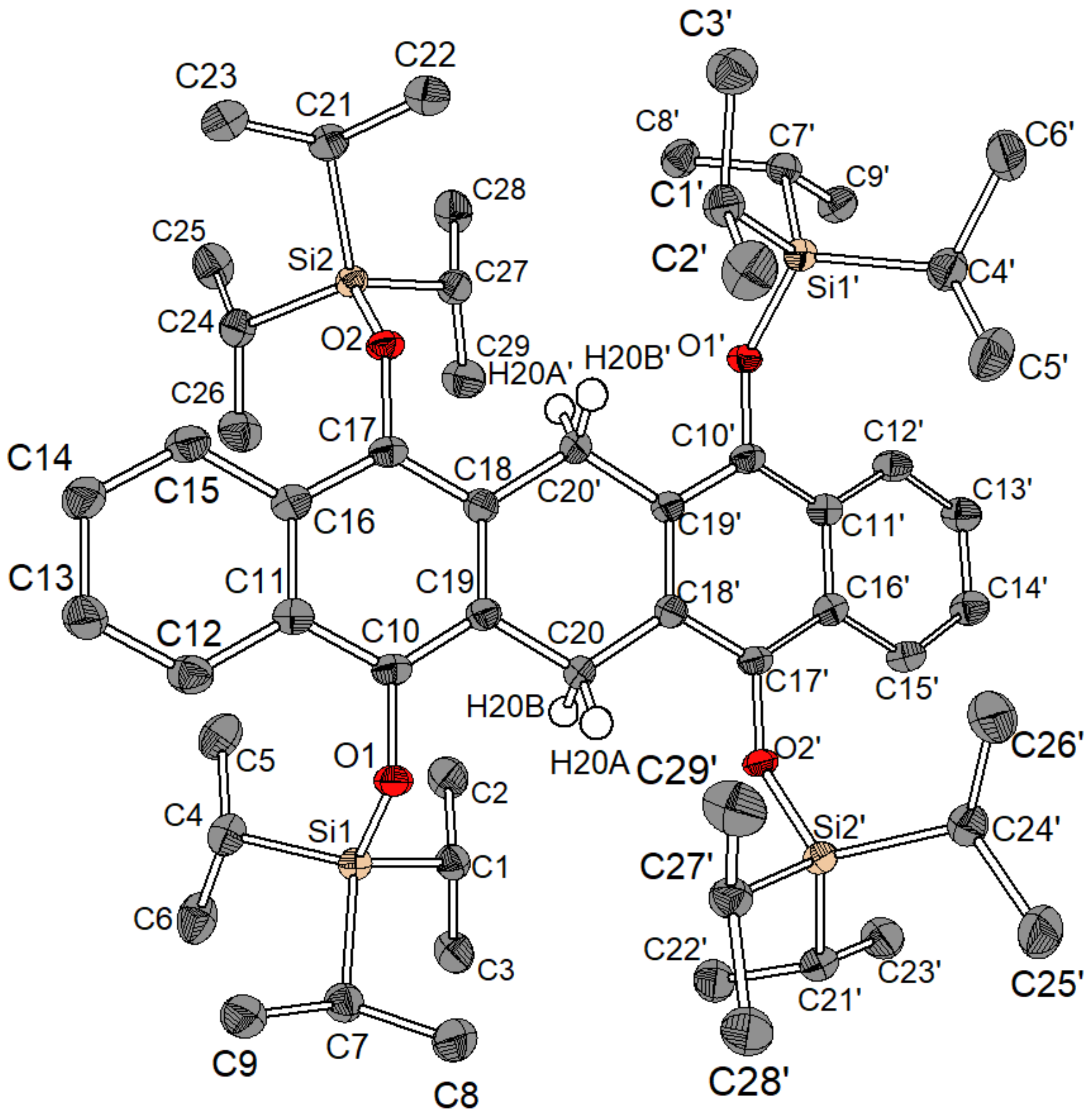

Figure S4: Solid state structures and packing of **10**. Some hydrogen atoms are omitted for clarity and thermal ellipsoids are shown at 50% probability level. Symmetry transformations: #1 -x+1,-y+1,-z+1

Table S2: Single crystal X-ray diffraction data for **9** and **10**.

Identification code	**9**	**10**
CCDC code	2257475	2257477
Empirical formula	C40 H52 O4 Si2	C58 H96 O4 Si4
Formula weight	652.99	969.70
Temperature	100(2) K	100(2) K
Wavelength	1.54178 Å	1.54178 Å
Crystal system	Triclinic	Triclinic
Space group	P -1	P -1
Unit cell dimensions	a = 8.2412(3) Å	a = 8.9080(2) Å
	b = 15.3699(5) Å	b = 12.7024(3) Å
	c = 17.2080(7) Å	c = 13.8339(4)Å
	α = 105.896(3)°	α= 66.670(2)°
	β = 93.829(3)°	β= 81.880(2)°
	γ = 105.392(3)°	γ = 88.080(2)°
Volume	1998.14(13) $Å^3$	1422.52(7) $Å^3$
Z	2	1
Density (calculated)	1.085 Mg/m^3	1.132 Mg/m^3
Absorption coefficient	1.080 mm^{-1}	1.292 mm^{-1}
F(000)	704	532
Crystal size	0.194 x 0.124 x 0.086 mm^3	0.146 x 0.114 x 0.099 mm^3
Theta range for data collection	4.716 to 66.567°.	3.514 to 69.347°
Index ranges	-9<=h<=9, -18<=k<=18, -20<=l<=11	-10<=h<=9, -15<=k<=14, -16<=l<=13
Reflections collected	35575	25192
Independent reflections	6967 [R(int) = 0.0673]	5211 [R(int) = 0.0556]
Completeness to theta = 66.567°	98.7%	98.9 %
Absorption correction	Semi-empirical from equivalents	Semi-empirical from equivalents
Max. and min. transmission	1.0000 and 0.3680	0.6765 and 0.5587
Refinement method	Full-matrix least-squares on F^2	Full-matrix least-squares on F^2
Data / restraints / parameters	6967 / 0 / 429	5211 / 0 / 310
Goodness-of-fit on F^2	1.051	1.092
Final R indices [I>2sigma(I)]	R1 = 0.0493, wR2 = 0.1300	R1 = 0.0384, wR2 = 0.1016
R indices (all data)	R1 = 0.0719, wR2 = 0.1412	R1 = 0.0515, wR2 = 0.1058
Extinction coefficient	n/a	n/a
Largest diff. peak and hole	0.367 and -0.346 $e.Å^{-3}$	0.274 and -0.284 $e.Å^{-3}$

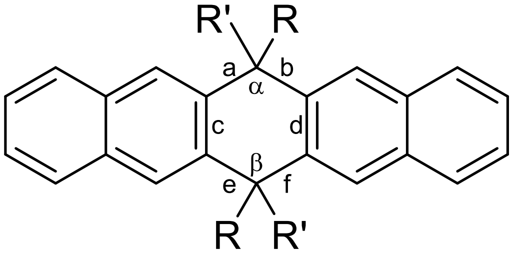

Table S3: Crystal Data of dihydro pentacenes and derivatives

R	R’	a	b	c	d	e	f	Source
H	H	1.5056(33)	1.5088(27)	1.4174(24)	1.4174(24)	1.5088(27)	1.5056(33)	**10**
H	H	1.5055(11)	1.5244(12)	1.4251(13)	1.4085(13)	1.5238(12)	1.4752(11)	[16]
H	H	1.5406(56)	1.5325(64)	1.4565(56)	1.4565(56)	1.5325(64)	1.5406(56)	[17]
OMe	Alkyne	1.5287(21)	1.5272(19)	1.4120(17)	1.4120(17)	1.5272(19)	1.5287(21)	[18]

R	R’	α	β	Source
H	H	117.3	117.3	**10**
H	H	113.7	112.4	[16]
H	H	117.3	117.3	[17]
OMe	Alkyne	114.3	114.3	[18]

Table S4: Bond length and angles of **7** and **8**.

7		8	
**Bond Length [Å]**		**Bond Length [Å]**	
C(1)-C(2)	1.363(4)	C(1)-O(1)	1.378(3)
C(1)-C(1)#1	1.421(5)	C(1)-C(2)	1.393(4)
C(1)-H(1)	0.9300	C(1)-C(22)	1.397(4)
C(2)-C(3)	1.432(3)	Cl(1)-C(25)	1.765(3)
C(2)-H(2)	0.9300	Cl(2)-C(25)	1.771(3)
C(3)-C(4)	1.396(3)	C(8)-O(2)	1.387(3)
C(3)-C(3)#1	1.434(4)	C(8)-C(7)	1.397(4)
C(4)-C(5)	1.393(3)	C(8)-C(9)	1.403(4)
C(4)-O(1)	1.400(3)	C(9)-C(10)	1.424(4)
C(5)-C(6)	1.425(3)	C(9)-C(22)	1.442(3)
C(5)-C(5)#1	1.445(4)	C(10)-C(11)	1.367(4)
C(6)-C(7)	1.368(3)	C(10)-H(10)	0.9300
C(6)-H(6)	0.9300	C(11)-C(20)	1.438(3)
C(7)-C(7)#1	1.438(4)	C(11)-C(12)	1.483(4)
C(7)-C(8)	1.483(3)	C(12)-O(3)	1.227(3)
C(8)-O(3)	1.233(3)	C(12)-C(13)	1.491(4)
C(8)-C(9)	1.491(3)	C(13)-C(14)	1.396(4)
C(9)-C(10)	1.392(3)	C(13)-C(18)	1.405(4)
C(9)-C(9)#1	1.397(4)	C(14)-C(15)	1.384(4)
C(10)-C(11)	1.391(4)	C(14)-H(14)	0.9300
C(10)-H(10)	0.9300	C(15)-C(16)	1.392(4)
C(11)-C(11)#1	1.389(5)	C(15)-H(15)	0.9300
C(11)-H(11)	0.9300	C(16)-C(17)	1.387(4)
C(12)-O(2)	1.197(3)	C(16)-H(16)	0.9300
C(12)-O(1)	1.376(3)	C(17)-C(18)	1.398(4)
C(12)-C(13)	1.490(3)	C(17)-H(17)	0.9300
C(13)-H(13A)	0.9600	C(18)-C(19)	1.493(4)
C(13)-H(13B)	0.9600	C(19)-O(4)	1.228(3)
C(13)-H(13C)	0.9600	C(19)-C(20)	1.487(3)
**Angles [°]**		C(20)-C(21)	1.369(4)
C(2)-C(1)-C(1)#1	120.59(15)	C(21)-C(22)	1.419(4)
C(2)-C(1)-H(1)	119.7	C(21)-H(21)	0.9300
C(1)#1-C(1)-H(1)	119.7	C(23)-O(1)	1.435(3)
C(1)-C(2)-C(3)	120.7(2)	C(23)-H(23A)	0.9600
C(1)-C(2)-H(2)	119.7	C(23)-H(23B)	0.9600
C(3)-C(2)-H(2)	119.7	C(23)-H(23C)	0.9600
C(4)-C(3)-C(2)	122.9(2)	C(24)-O(2)	1.434(3)
C(4)-C(3)-C(3)#1	118.45(14)	C(24)-H(24A)	0.9600
C(2)-C(3)-C(3)#1	118.69(14)	C(24)-H(24B)	0.9600
C(5)-C(4)-C(3)	123.3(2)	C(24)-H(24C)	0.9600
C(5)-C(4)-O(1)	120.1(2)	C(25)-H(25A)	0.9700
C(3)-C(4)-O(1)	116.4(2)	C(25)-H(25B)	0.9700
C(4)-C(5)-C(6)	123.0(2)	C(2)-C(3)	1.423(4)
C(4)-C(5)-C(5)#1	118.27(14)	C(2)-C(7)	1.445(4)
C(6)-C(5)-C(5)#1	118.75(13)	C(3)-C(4)	1.362(4)
C(7)-C(6)-C(5)	120.9(2)	C(3)-H(3)	0.9300
C(7)-C(6)-H(6)	119.5	C(4)-C(5)	1.425(4)
C(5)-C(6)-H(6)	119.5	C(4)-H(4)	0.9300
C(6)-C(7)-C(7)#1	120.20(14)	C(5)-C(6)	1.355(4)
C(6)-C(7)-C(8)	119.2(2)	C(5)-H(5)	0.9300
C(7)#1-C(7)-C(8)	120.59(13)	C(6)-C(7)	1.430(4)
O(3)-C(8)-C(7)	121.6(2)	C(6)-H(6)	0.9300
O(3)-C(8)-C(9)	120.7(2)	**Angles [°]**	
C(7)-C(8)-C(9)	117.7(2)	O(1)-C(1)-C(2)	119.3(2)
C(10)-C(9)-C(9)#1	119.67(14)	O(1)-C(1)-C(22)	118.8(2)
C(10)-C(9)-C(8)	118.8(2)	C(2)-C(1)-C(22)	121.8(2)
C(9)#1-C(9)-C(8)	121.47(13)	O(2)-C(8)-C(7)	118.9(2)
C(11)-C(10)-C(9)	120.4(2)	O(2)-C(8)-C(9)	119.5(2)
C(11)-C(10)-H(10)	119.8	C(7)-C(8)-C(9)	121.5(2)
C(9)-C(10)-H(10)	119.8	C(8)-C(9)-C(10)	122.6(2)
C(11)#1-C(11)-C(10)	119.89(15)	C(8)-C(9)-C(22)	119.1(2)
C(11)#1-C(11)-H(11)	120.1	C(10)-C(9)-C(22)	118.3(2)
C(10)-C(11)-H(11)	120.1	C(11)-C(10)-C(9)	121.4(2)
O(2)-C(12)-O(1)	122.8(2)	C(11)-C(10)-H(10)	119.3
O(2)-C(12)-C(13)	127.8(2)	C(9)-C(10)-H(10)	119.3

O(1)-C(12)-C(13)	109.3(2)	C(10)-C(11)-C(20)	120.2(2)
C(12)-C(13)-H(13A)	109.5	C(10)-C(11)-C(12)	119.4(2)
C(12)-C(13)-H(13B)	109.5	C(20)-C(11)-C(12)	120.4(2)
H(13A)-C(13)-H(13B)	109.5	O(3)-C(12)-C(11)	121.5(2)
C(12)-C(13)-H(13C)	109.5	O(3)-C(12)-C(13)	120.4(3)
H(13A)-C(13)-H(13C)	109.5	C(11)-C(12)-C(13)	118.0(2)
H(13B)-C(13)-H(13C)	109.5	C(14)-C(13)-C(18)	119.0(3)
C(12)-O(1)-C(4)	119.41(18)	C(14)-C(13)-C(12)	119.4(3)
**Symmetry transformations used to generate equivalent atoms**		C(18)-C(13)-C(12)	121.6(2)
#1 -x+1,y,-z+1/2		C(15)-C(14)-C(13)	120.7(3)
		C(15)-C(14)-H(14)	119.7
		C(13)-C(14)-H(14)	119.7
		C(14)-C(15)-C(16)	120.3(3)
		C(14)-C(15)-H(15)	119.9
		C(16)-C(15)-H(15)	119.9
		C(17)-C(16)-C(15)	119.9(3)
		C(17)-C(16)-H(16)	120.0
		C(15)-C(16)-H(16)	120.0
		C(16)-C(17)-C(18)	120.1(3)
		C(16)-C(17)-H(17)	119.9
		C(18)-C(17)-H(17)	119.9
		C(17)-C(18)-C(13)	120.0(3)
		C(17)-C(18)-C(19)	118.9(2)
		C(13)-C(18)-C(19)	121.0(2)
		O(4)-C(19)-C(20)	121.5(3)
		O(4)-C(19)-C(18)	120.7(2)
		C(20)-C(19)-C(18)	117.8(2)
		C(21)-C(20)-C(11)	119.7(2)
		C(21)-C(20)-C(19)	119.2(2)
		C(11)-C(20)-C(19)	121.1(2)
		C(20)-C(21)-C(22)	121.5(2)
		C(20)-C(21)-H(21)	119.3
		C(22)-C(21)-H(21)	119.3
		C(1)-C(22)-C(21)	121.9(2)
		C(1)-C(22)-C(9)	119.2(2)
		C(21)-C(22)-C(9)	118.9(2)
		O(1)-C(23)-H(23A)	109.5
		O(1)-C(23)-H(23B)	109.5
		H(23A)-C(23)-H(23B)	109.5
		O(1)-C(23)-H(23C)	109.5
		H(23A)-C(23)-H(23C)	109.5
		H(23B)-C(23)-H(23C)	109.5
		O(2)-C(24)-H(24A)	109.5
		O(2)-C(24)-H(24B)	109.5
		H(24A)-C(24)-H(24B)	109.5
		O(2)-C(24)-H(24C)	109.5
		H(24A)-C(24)-H(24C)	109.5
		H(24B)-C(24)-H(24C)	109.5
		Cl(1)-C(25)-Cl(2)	111.16(17)
		Cl(1)-C(25)-H(25A)	109.4
		Cl(2)-C(25)-H(25A)	109.4
		Cl(1)-C(25)-H(25B)	109.4
		Cl(2)-C(25)-H(25B)	109.4
		H(25A)-C(25)-H(25B)	108.0
		C(1)-O(1)-C(23)	113.3(2)
		C(8)-O(2)-C(24)	114.6(2)
		C(1)-C(2)-C(3)	122.1(3)
		C(1)-C(2)-C(7)	119.2(2)
		C(3)-C(2)-C(7)	118.7(2)
		C(4)-C(3)-C(2)	121.1(3)
		C(4)-C(3)-H(3)	119.4
		C(2)-C(3)-H(3)	119.4
		C(3)-C(4)-C(5)	119.9(3)
		C(3)-C(4)-H(4)	120.0
		C(5)-C(4)-H(4)	120.0
		C(6)-C(5)-C(4)	121.3(3)
		C(6)-C(5)-H(5)	119.4
		C(4)-C(5)-H(5)	119.4

		C(5)-C(6)-C(7)	120.6(3)
		C(5)-C(6)-H(6)	119.7
		C(7)-C(6)-H(6)	119.7
		C(8)-C(7)-C(6)	122.5(2)
		C(8)-C(7)-C(2)	119.2(2)
		C(6)-C(7)-C(2)	118.4(2)

Table S5: Bond length and angles of **9** and **10**.

**9**		**10**	
**Bond Length [Å]**		**Bond Length [Å]**	
C(1)-O(1)	1.363(3)	C(1)-C(3)	1.540(3)
C(1)-C(22)	1.398(3)	C(1)-C(2)	1.546(3)
C(1)-C(2)	1.412(3)	C(1)-Si(1)	1.8856(18)
C(2)-C(3)	1.420(3)	C(4)-C(5)	1.537(3)
C(2)-C(7)	1.434(3)	C(4)-C(6)	1.540(3)
C(3)-C(4)	1.366(3)	C(4)-Si(1)	1.8911(19)
C(4)-C(5)	1.420(3)	C(7)-C(8)	1.531(3)
C(5)-C(6)	1.359(3)	C(7)-C(9)	1.537(2)
C(6)-C(7)	1.430(3)	C(7)-Si(1)	1.8830(18)
C(7)-C(8)	1.408(3)	C(10)-O(1)	1.382(2)
C(8)-O(2)	1.356(3)	C(10)-C(19)	1.383(2)
C(8)-C(9)	1.404(3)	C(10)-C(11)	1.416(2)
C(9)-C(10)	1.424(3)	C(11)-C(16)	1.421(2)
C(9)-C(22)	1.439(3)	C(11)-C(12)	1.424(2)
C(10)-C(11)	1.367(3)	C(12)-C(13)	1.365(3)
C(11)-C(20)	1.433(3)	C(13)-C(14)	1.408(3)
C(11)-C(12)	1.490(3)	C(14)-C(15)	1.372(2)
C(12)-O(3)	1.209(3)	C(15)-C(16)	1.415(2)
C(12)-C(13)	1.497(3)	C(16)-C(17)	1.423(2)
C(13)-C(18)	1.397(3)	C(17)-O(2)	1.3786(19)
C(13)-C(14)	1.402(3)	C(17)-C(18)	1.379(2)
C(14)-C(15)	1.383(3)	C(18)-C(19)	1.417(2)
C(15)-C(16)	1.386(4)	C(18)-C(20)#1	1.506(2)
C(16)-C(17)	1.382(4)	C(19)-C(20)	1.509(2)
C(17)-C(18)	1.404(3)	C(21)-C(23)	1.533(3)
C(18)-C(19)	1.493(3)	C(21)-C(22)	1.535(3)
C(19)-O(4)	1.218(3)	C(21)-Si(2)	1.8832(19)
C(19)-C(20)	1.486(3)	C(24)-C(26)	1.536(3)
C(20)-C(21)	1.367(3)	C(24)-C(25)	1.542(3)
C(21)-C(22)	1.421(3)	C(24)-Si(2)	1.8890(19)
C(23)-C(24)	1.533(4)	C(27)-C(28)	1.533(2)
C(23)-C(25)	1.547(3)	C(27)-C(29)	1.539(3)
C(23)-Si(1)	1.876(2)	C(27)-Si(2)	1.8839(18)
C(26)-C(28)	1.539(4)	O(1)-Si(1)	1.6709(12)
C(26)-C(27)	1.540(3)	O(2)-Si(2)	1.6660(12)
C(26)-Si(1)	1.880(3)	**Angles [°]**	
C(29)-C(31)	1.540(3)	C(3)-C(1)-C(2)	109.55(17)
C(29)-C(30)	1.543(4)	C(3)-C(1)-Si(1)	114.68(12)
C(29)-Si(1)	1.875(3)	C(2)-C(1)-Si(1)	113.87(13)
C(32)-C(33)	1.542(4)	C(5)-C(4)-C(6)	110.44(17)
C(32)-C(34)	1.544(3)	C(5)-C(4)-Si(1)	117.60(13)
C(32)-Si(2)	1.880(2)	C(6)-C(4)-Si(1)	109.31(13)
C(35)-C(36)	1.534(3)	C(8)-C(7)-C(9)	109.93(16)
C(35)-C(37)	1.545(3)	C(8)-C(7)-Si(1)	112.54(12)
C(35)-Si(2)	1.877(2)	C(9)-C(7)-Si(1)	111.56(12)
C(38)-C(40)	1.531(4)	O(1)-C(10)-C(19)	119.64(15)
C(38)-C(39)	1.552(3)	O(1)-C(10)-C(11)	118.92(14)
C(38)-Si(2)	1.881(3)	C(19)-C(10)-C(11)	121.23(15)
O(1)-Si(1)	1.6855(16)	C(10)-C(11)-C(16)	118.88(15)
O(2)-Si(2)	1.6874(16)	C(10)-C(11)-C(12)	122.72(16)

**Angles [°]**		C(16)-C(11)-C(12)	118.39(16)
O(1)-C(1)-C(22)	120.8(2)	C(13)-C(12)-C(11)	121.09(16)
O(1)-C(1)-C(2)	118.6(2)	C(12)-C(13)-C(14)	120.51(16)
C(22)-C(1)-C(2)	120.5(2)	C(15)-C(14)-C(13)	119.78(17)
C(1)-C(2)-C(3)	121.9(2)	C(14)-C(15)-C(16)	121.29(16)
C(1)-C(2)-C(7)	119.2(2)	C(15)-C(16)-C(11)	118.85(15)
C(3)-C(2)-C(7)	119.0(2)	C(15)-C(16)-C(17)	122.60(15)
C(4)-C(3)-C(2)	120.9(2)	C(11)-C(16)-C(17)	118.54(16)
C(3)-C(4)-C(5)	120.3(2)	O(2)-C(17)-C(18)	120.17(15)
C(6)-C(5)-C(4)	120.6(2)	O(2)-C(17)-C(16)	118.34(15)
C(5)-C(6)-C(7)	120.9(2)	C(18)-C(17)-C(16)	121.21(15)
C(8)-C(7)-C(6)	121.3(2)	C(17)-C(18)-C(19)	119.78(15)
C(8)-C(7)-C(2)	120.4(2)	C(17)-C(18)-C(20)#1	118.88(15)
C(6)-C(7)-C(2)	118.3(2)	C(19)-C(18)-C(20)#1	121.27(16)
O(2)-C(8)-C(9)	120.7(2)	C(10)-C(19)-C(18)	119.61(16)
O(2)-C(8)-C(7)	119.1(2)	C(10)-C(19)-C(20)	119.03(15)
C(9)-C(8)-C(7)	120.1(2)	C(18)-C(19)-C(20)	121.36(15)
C(8)-C(9)-C(10)	121.7(2)	C(18)#1-C(20)-C(19)	117.25(15)
C(8)-C(9)-C(22)	119.7(2)	C(23)-C(21)-C(22)	109.60(16)
C(10)-C(9)-C(22)	118.6(2)	C(23)-C(21)-Si(2)	112.89(12)
C(11)-C(10)-C(9)	121.6(2)	C(22)-C(21)-Si(2)	111.46(12)
C(10)-C(11)-C(20)	119.8(2)	C(26)-C(24)-C(25)	109.71(17)
C(10)-C(11)-C(12)	118.8(2)	C(26)-C(24)-Si(2)	117.41(13)
C(20)-C(11)-C(12)	121.4(2)	C(25)-C(24)-Si(2)	110.35(12)
O(3)-C(12)-C(11)	122.0(2)	C(28)-C(27)-C(29)	110.22(17)
O(3)-C(12)-C(13)	121.6(2)	C(28)-C(27)-Si(2)	115.29(13)
C(11)-C(12)-C(13)	116.4(2)	C(29)-C(27)-Si(2)	113.17(12)
C(18)-C(13)-C(14)	119.4(2)	C(10)-O(1)-Si(1)	134.20(11)
C(18)-C(13)-C(12)	122.3(2)	C(17)-O(2)-Si(2)	134.39(11)
C(14)-C(13)-C(12)	118.3(2)	O(1)-Si(1)-C(7)	102.17(7)
C(15)-C(14)-C(13)	119.8(2)	O(1)-Si(1)-C(1)	108.48(7)
C(14)-C(15)-C(16)	120.6(2)	C(7)-Si(1)-C(1)	110.99(8)
C(17)-C(16)-C(15)	120.6(2)	O(1)-Si(1)-C(4)	112.70(8)
C(16)-C(17)-C(18)	119.3(2)	C(7)-Si(1)-C(4)	107.76(8)
C(13)-C(18)-C(17)	120.3(2)	C(1)-Si(1)-C(4)	114.11(9)
C(13)-C(18)-C(19)	121.6(2)	O(2)-Si(2)-C(21)	102.30(7)
C(17)-C(18)-C(19)	118.1(2)	O(2)-Si(2)-C(27)	108.04(7)
O(4)-C(19)-C(20)	121.3(2)	C(21)-Si(2)-C(27)	109.90(8)
O(4)-C(19)-C(18)	121.7(2)	O(2)-Si(2)-C(24)	112.37(7)
C(20)-C(19)-C(18)	117.1(2)	C(21)-Si(2)-C(24)	109.86(8)
C(21)-C(20)-C(11)	119.9(2)	C(27)-Si(2)-C(24)	113.75(9)
C(21)-C(20)-C(19)	118.9(2)	**Symmetry transformations used to generate equivalent atoms**	
C(11)-C(20)-C(19)	121.2(2)	#1 -x+1,-y+1,-z+1	
C(20)-C(21)-C(22)	121.8(2)		
C(1)-C(22)-C(21)	121.7(2)		
C(1)-C(22)-C(9)	120.0(2)		
C(21)-C(22)-C(9)	118.3(2)		
C(24)-C(23)-C(25)	110.8(2)		
C(24)-C(23)-Si(1)	113.67(18)		
C(25)-C(23)-Si(1)	114.83(18)		
C(28)-C(26)-C(27)	110.5(2)		
C(28)-C(26)-Si(1)	112.84(19)		
C(27)-C(26)-Si(1)	116.13(18)		
C(31)-C(29)-C(30)	110.1(2)		
C(31)-C(29)-Si(1)	113.31(19)		
C(30)-C(29)-Si(1)	113.70(18)		
C(33)-C(32)-C(34)	110.8(2)		
C(33)-C(32)-Si(2)	113.10(17)		
C(34)-C(32)-Si(2)	114.13(17)		
C(36)-C(35)-C(37)	110.4(2)		
C(36)-C(35)-Si(2)	115.76(17)		

C(37)-C(35)-Si(2)	112.48(18)		
C(40)-C(38)-C(39)	110.3(2)		
C(40)-C(38)-Si(2)	113.63(18)		
C(39)-C(38)-Si(2)	112.88(19)		
C(1)-O(1)-Si(1)	132.18(15)		
C(8)-O(2)-Si(2)	130.59(14)		
O(1)-Si(1)-C(29)	105.97(10)		
O(1)-Si(1)-C(23)	108.15(10)		
C(29)-Si(1)-C(23)	110.99(12)		
O(1)-Si(1)-C(26)	103.39(10)		
C(29)-Si(1)-C(26)	115.55(12)		
C(23)-Si(1)-C(26)	112.07(12)		
O(2)-Si(2)-C(35)	104.31(10)		
O(2)-Si(2)-C(32)	107.09(10)		
C(35)-Si(2)-C(32)	111.74(12)		
O(2)-Si(2)-C(38)	106.05(10)		
C(35)-Si(2)-C(38)	115.32(12)		
C(32)-Si(2)-C(38)	111.56(12)		

A relaxed potential energy surface scan was performed for compound **10** in the gas phase on the B3LYP/def2-svp level of theory with the D3 version of Grimme's dispersion model with the original D3 damping function, using the gaussian 16 program suite.[10,19–21] The angle C12-C20-C15' was varied stepwise. An angle of 180° corresponds to a planar pentacyclic core, while deviations from 180° lead to a U-shaped bending about the axis connecting the two CH_2 carbon atoms of the central six-membered ring. Two OTIPS groups bound to one of the six-membered rings point towards the concave side of the U-shaped core, while the two others point towards the convex side. A local minimum was estimated at an angle of ca. 143° in good congruency with the results (139°) from a free geometry optimization. A maximum is found at an angle around 180°, which is only 3.3 kcal/mol higher in energy in our computational model. This indicates a low barrier for the bending of compound **10** about the axis that connects the two CH_2 carbon atoms of the central six-membered ring and supports packing effects to account for the stabilization of the planar conformer of compound **10** in the solid state.

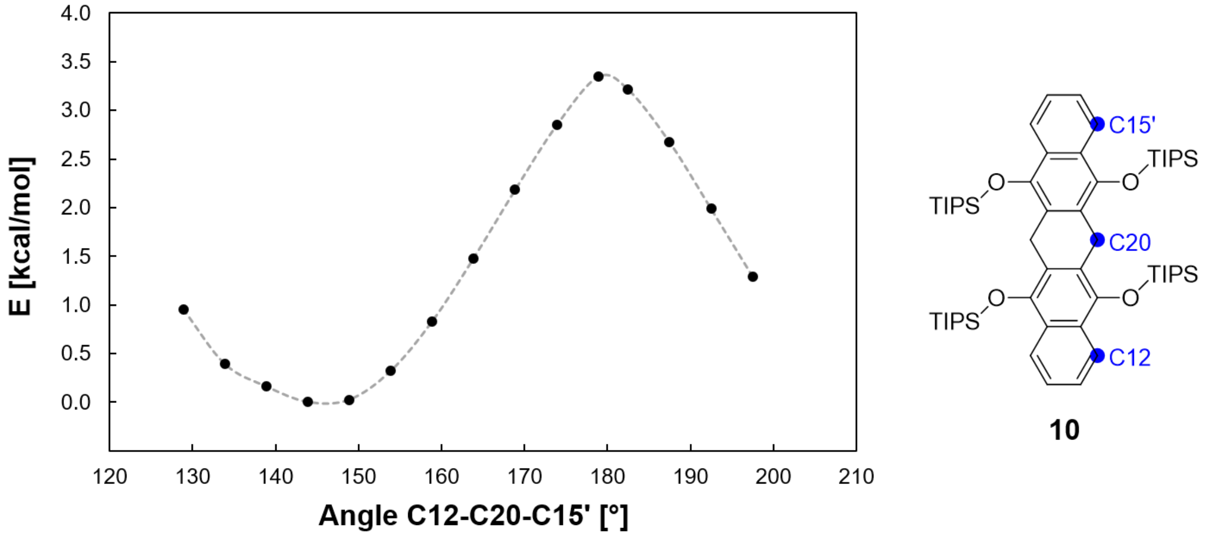

10

Figure S5: Energy diagram showing the results of a relaxed potential energy surface scan of compound **10** in the gas phase with stepwise variations of the C12-C20-C15' angle. An angle C12-C20-C15' of 180° corresponds to a planar pentacyclic core, while deviations from 180° lead to a U-shaped bending about the axis connecting the two CH_2 carbon atoms of the central six-membered ring. E = total energy.

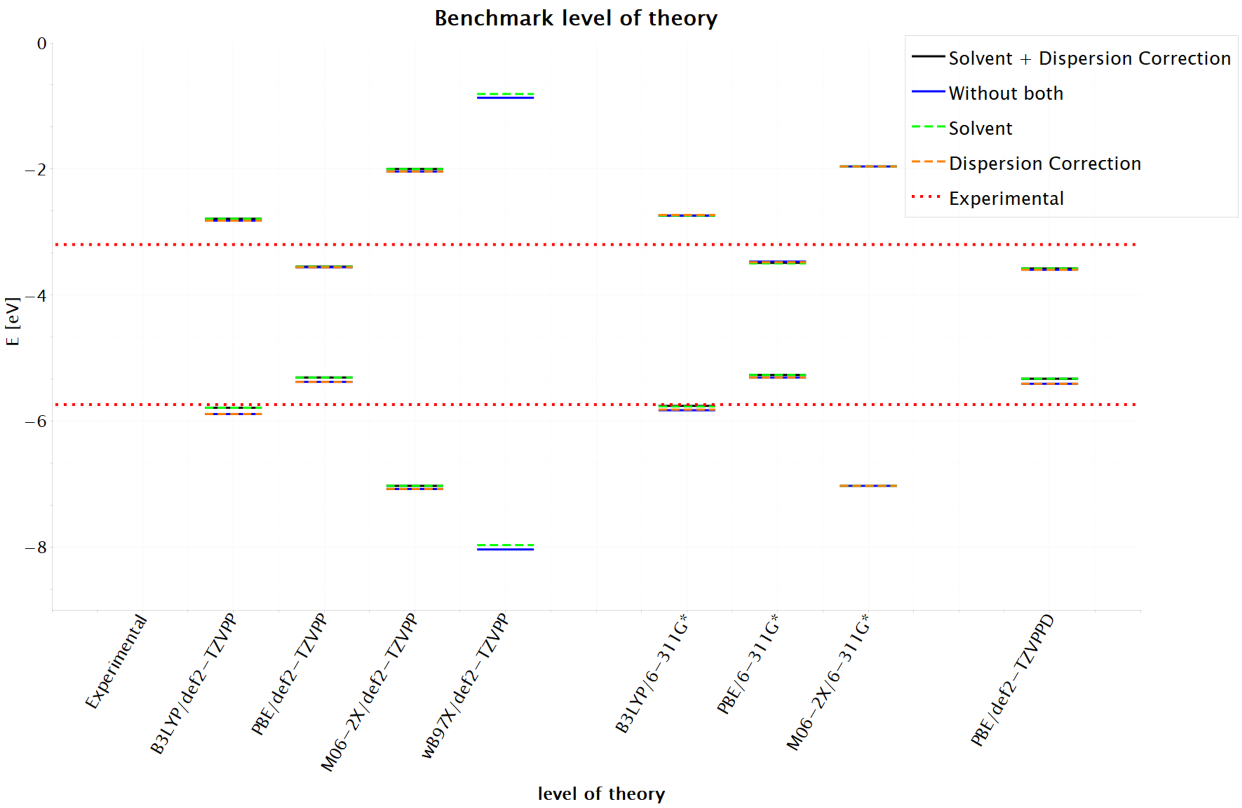

Figure S6: Calculation of the HOMO and LUMO energy of Acetate **7** using different functionals and basis sets with and without dispersion correction and consideration of solvent. The data are compared to the experimental obtained results.

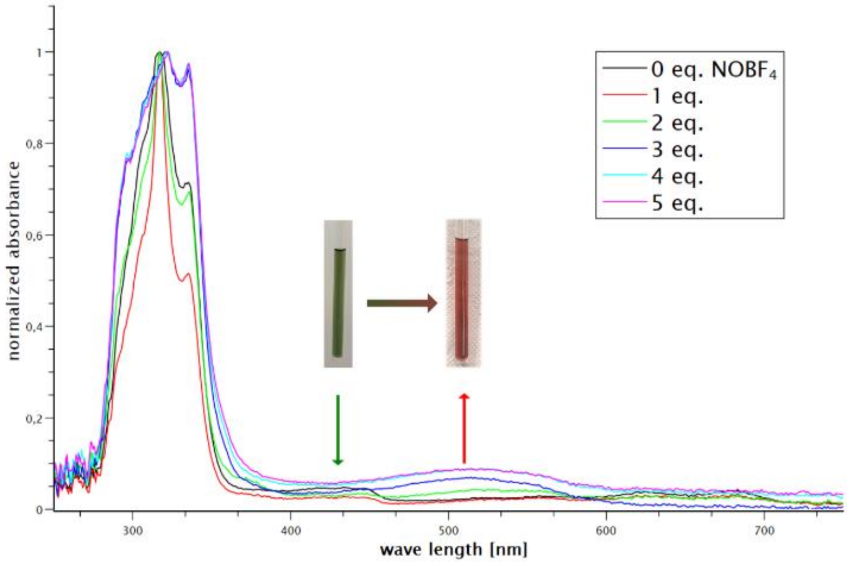

Figure S7: UV/Vis-spectrum of 10 (in dichloromethane) in a titration experiment with $NOBF_4$ (in acetonitrile) from 0 eq. to 5 eq. The color changed from green to red.

NMR spectra

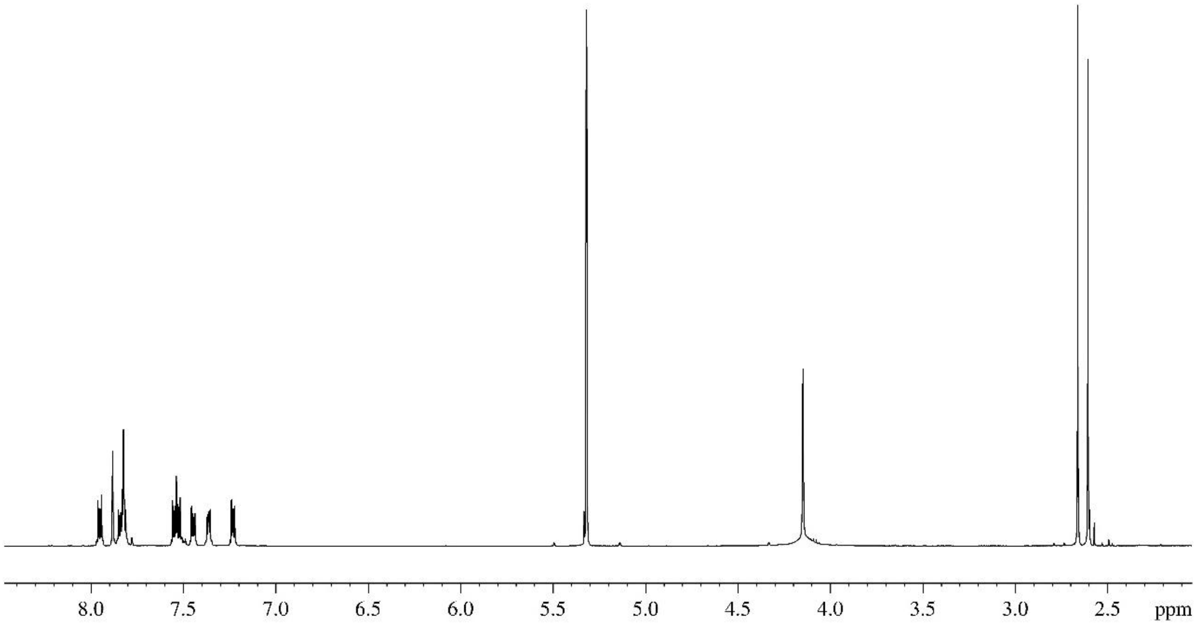

Figure S8: ^1H NMR (500 MHz, 300 K, CD_2Cl_2) of the isolated product mixture of the reduction of **6** with Zn and NaOAc in acetyl anhydride.

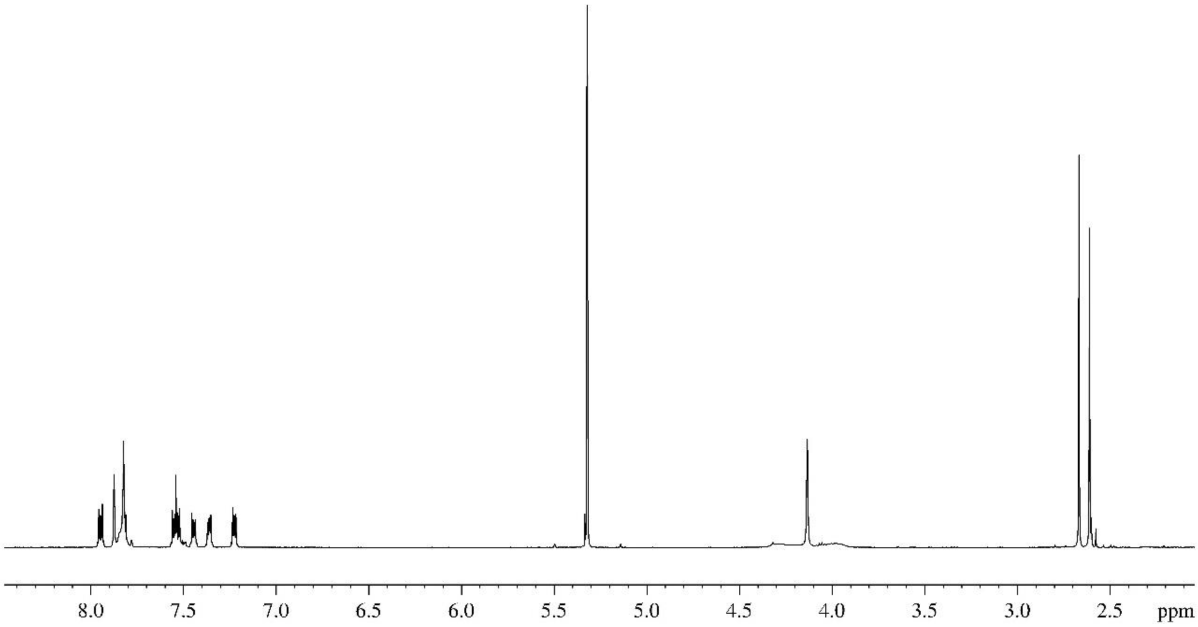

Figure S9: ^1H NMR (500 MHz CD_2Cl_2) of the isolated product mixture of the reduction of **6** with Zn and NaOAc in acetyl anhydride at 260 K, showing coalescence of the split CH_2 signals 3.9 – 4.3 ppm.

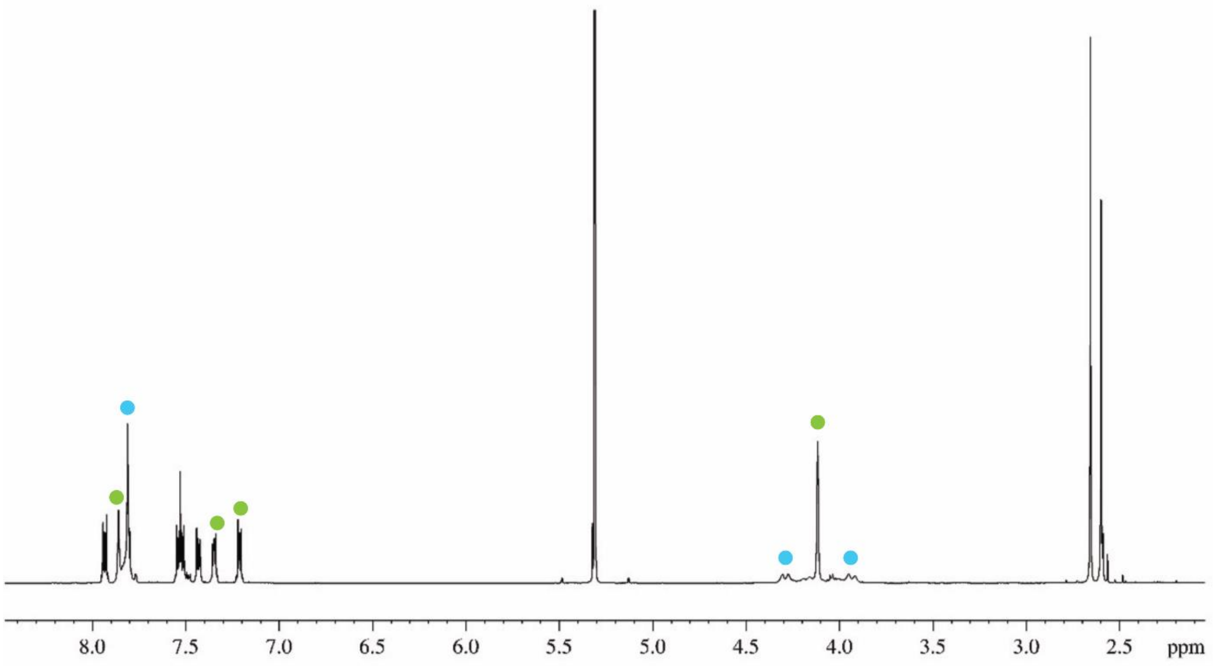

Figure S10: ^1H NMR (500 MHz, 253 K, CD_2Cl_2) of the isolated product mixture of the reduction of **6** with Zn and NaOAc in acetyl anhydride. The color indicated signals can be assigned to the corresponding methylene signals.

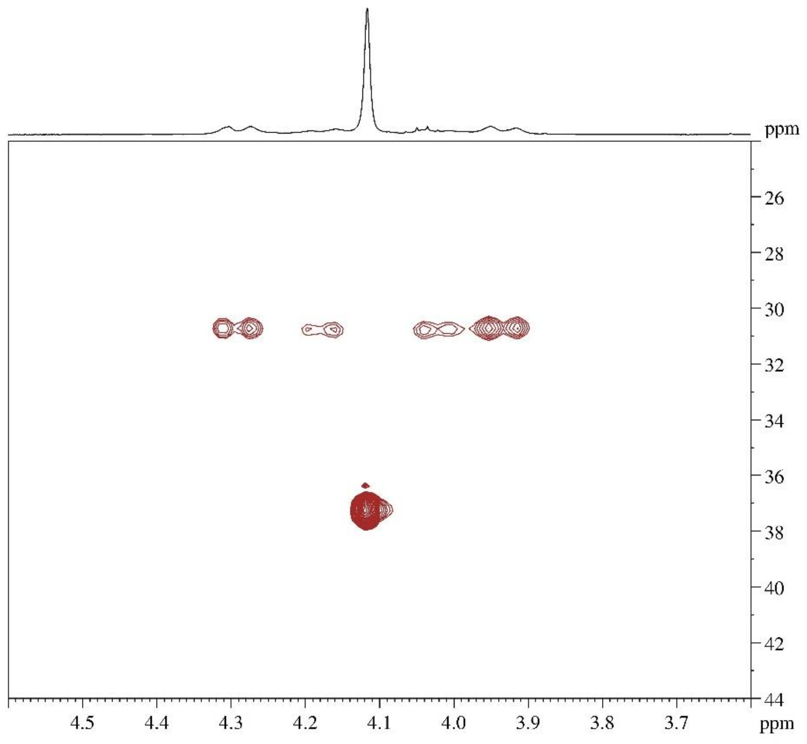

Figure S11: Section of ^1H, ^{13}C HSQC spectrum (500 MHz, 253 K, CD_2Cl_2) of the isolated product mixture of the reduction of **6** with Zn and NaOAc in acetyl anhydride showing cross peaks of the split (upper trace) and the non-split (lower trace) ^1H signals of the methylene groups in the sample.

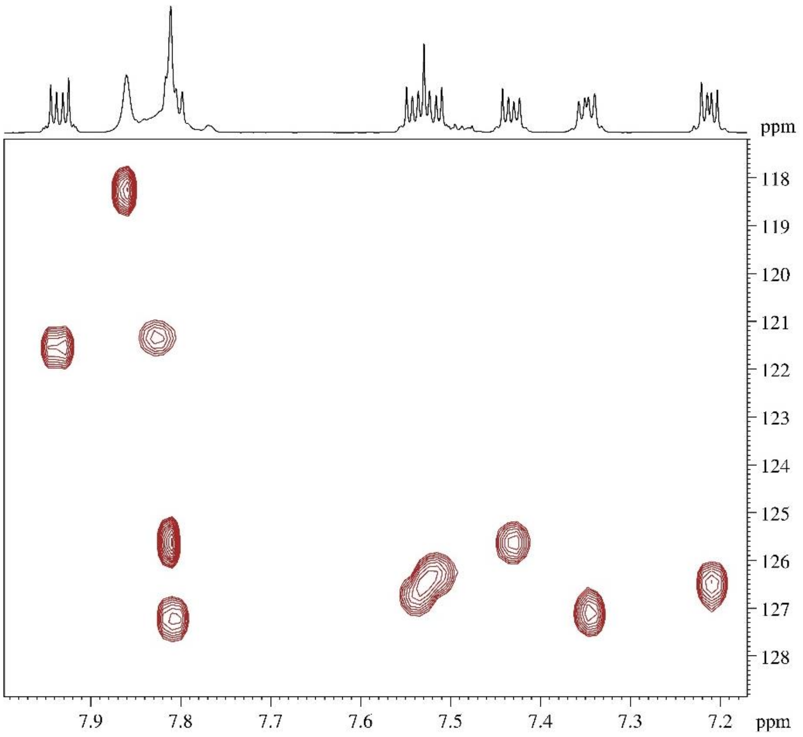

Figure S12: Section of ^1H, ^{13}C HSQC spectrum (500 MHz, 253 K, CD_2Cl_2) of the isolated product mixture of the reduction of **6** with Zn and NaOAc in acetyl anhydride showing cross peaks of 10 different aromatic CH groups in the sample.

Estimation of free energy of activation by variable temperature ^1H NMR:

At room temperature a singlet was observed, whereas at 190 K split doublet of doublets were detected. A difference in resonance frequencies $\delta\nu$ = 208 Hz was measured at 190 K. Coalescence of the split signals were detected at 260 K. Therefore, an exchange rate at coalescence k_{coal} (= 2.22 $\delta\nu$) of 462 Hz was obtained.[22] Furthermore, we estimated the free energy of the activation $\Delta G^{\ddagger}$ by using the approximate solution to Eyring equation as follows: $\Delta G^{\ddagger} = R\,T_c\,[22.96 + \ln(T_c / \delta\nu)]$ [J/mol], where R is the universal gas constant (8.315 J K^{-1} mol^{-1}) and T_c the coalescence temperature. In this way the free energy of the activation $\Delta G^{\ddagger}$ was estimated to be about 12 kcal/mol.

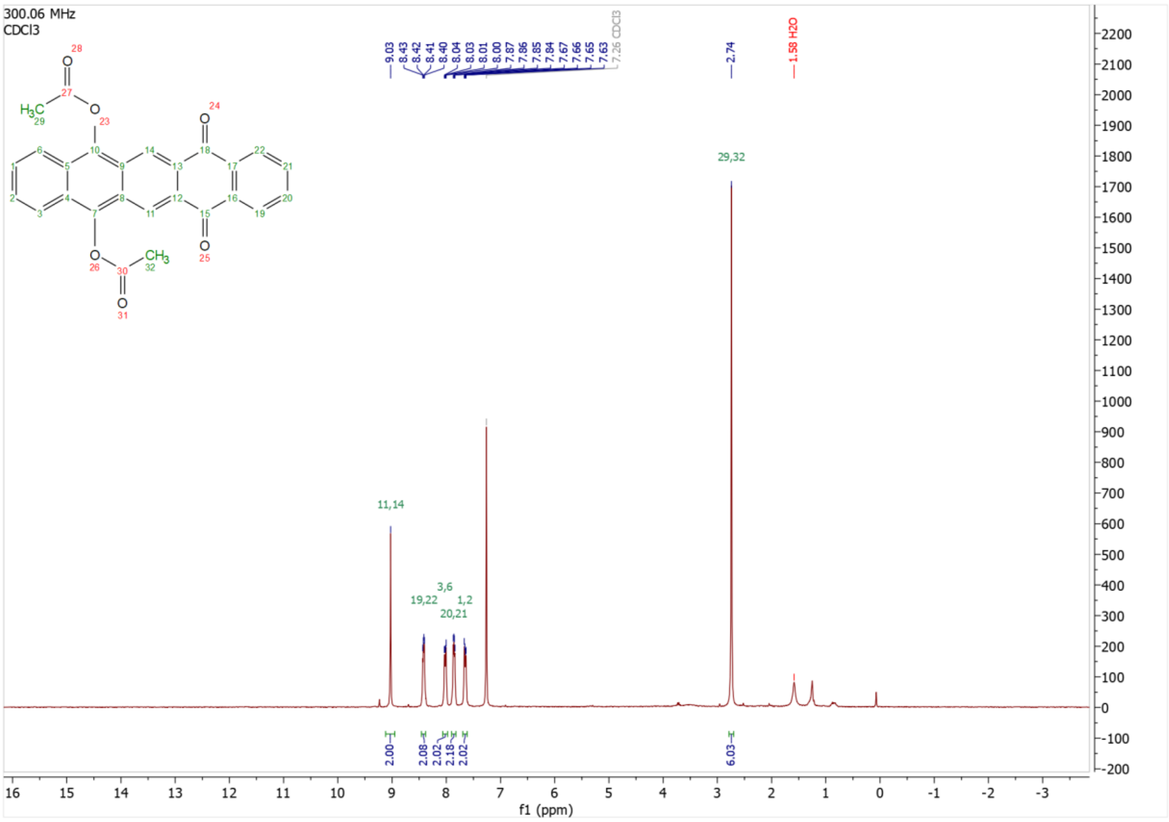

Figure S13: ^{1}H NMR (300 MHz, 298 K, $CDCl_3$) of **7**.

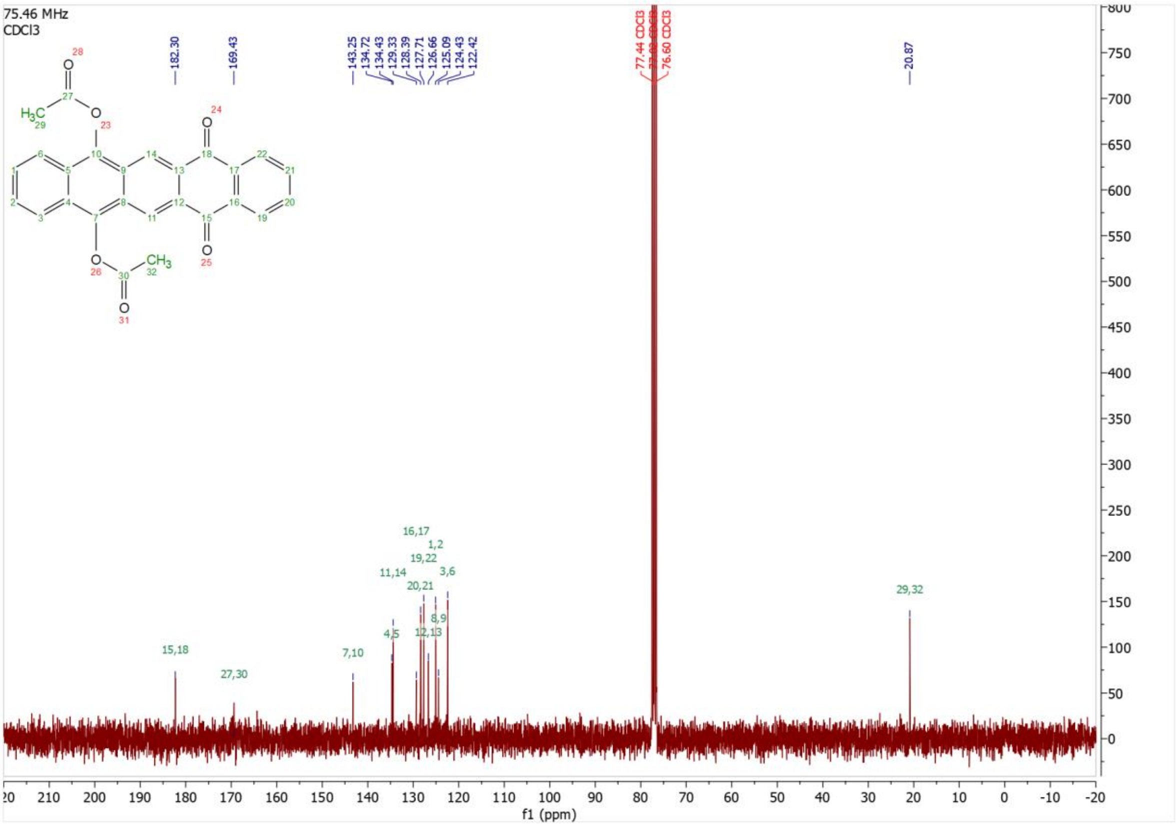

Figure S14: $^{13}C\{^{1}H\}$ NMR (300 MHz, 298 K, $CDCl_3$) of **7**.

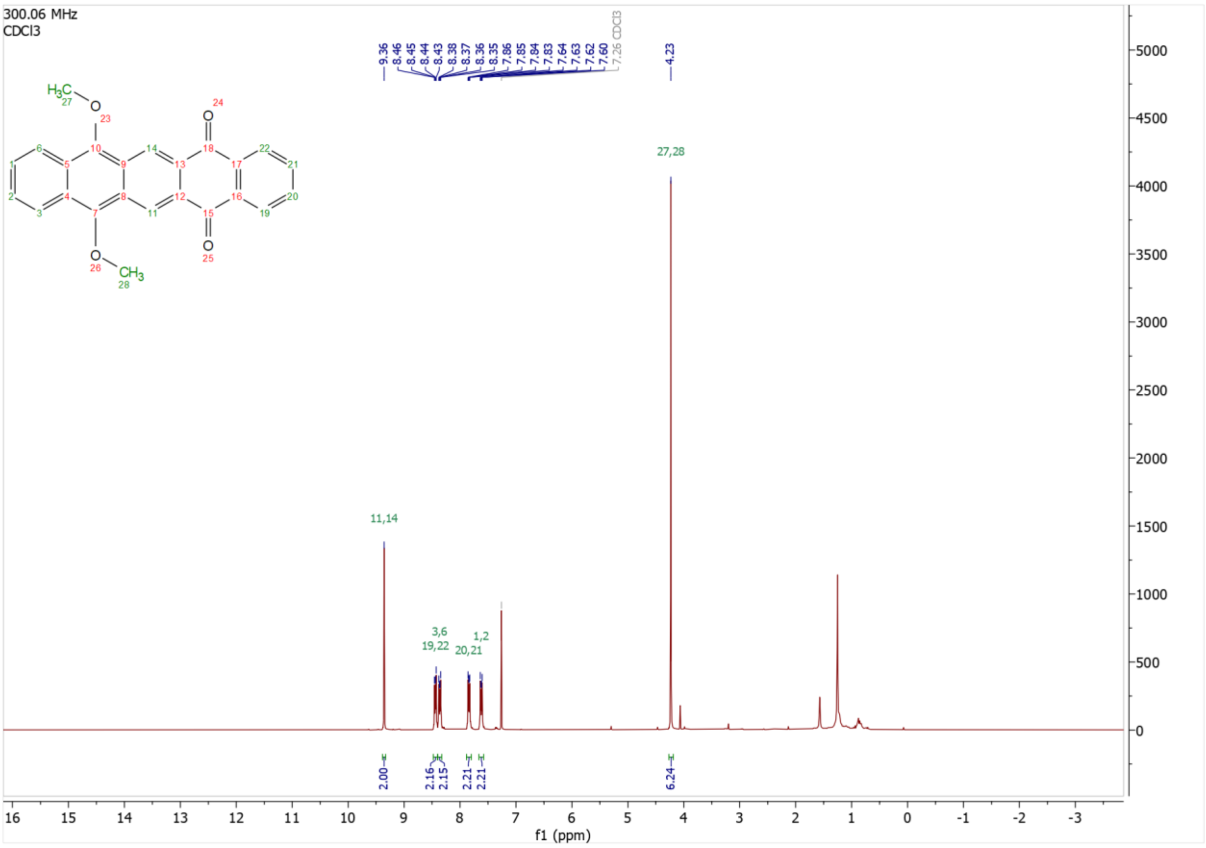

Figure S15: ^{1}H NMR (300 MHz, 298 K, $CDCl_3$) of **8**.

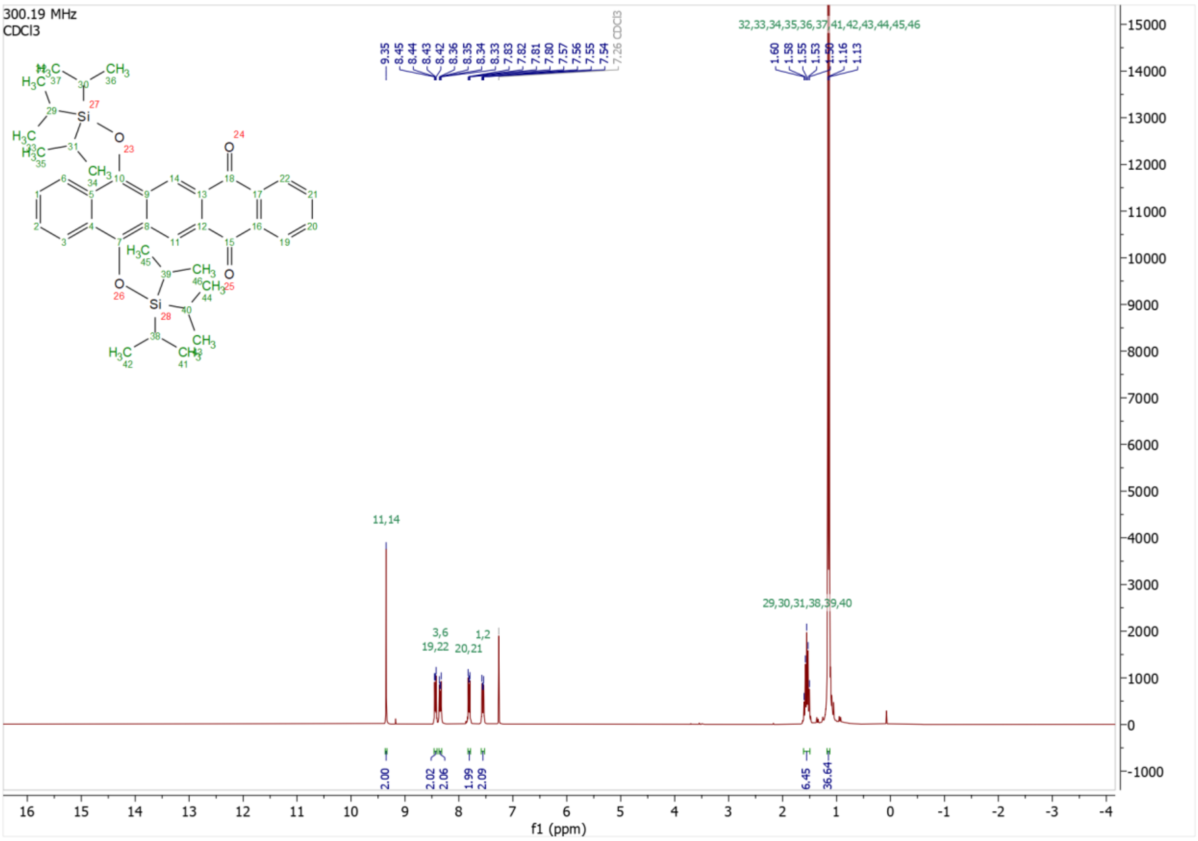

Figure S16: ^{1}H NMR (300 MHz, 298 K, $CDCl_3$) of **9**.

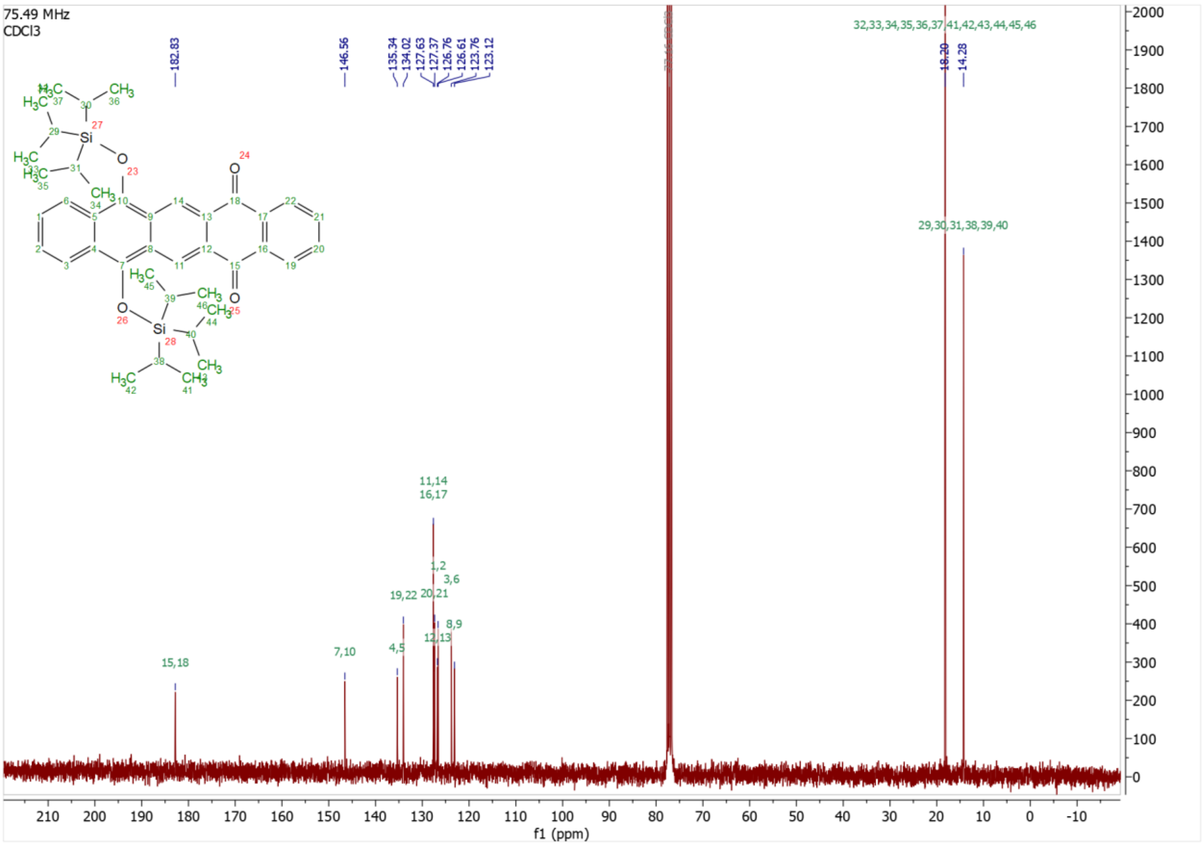

Figure S17: $^{13}C\{^{1}H\}$ NMR (300 MHz, 298 K, $CDCl_3$) of **9**.

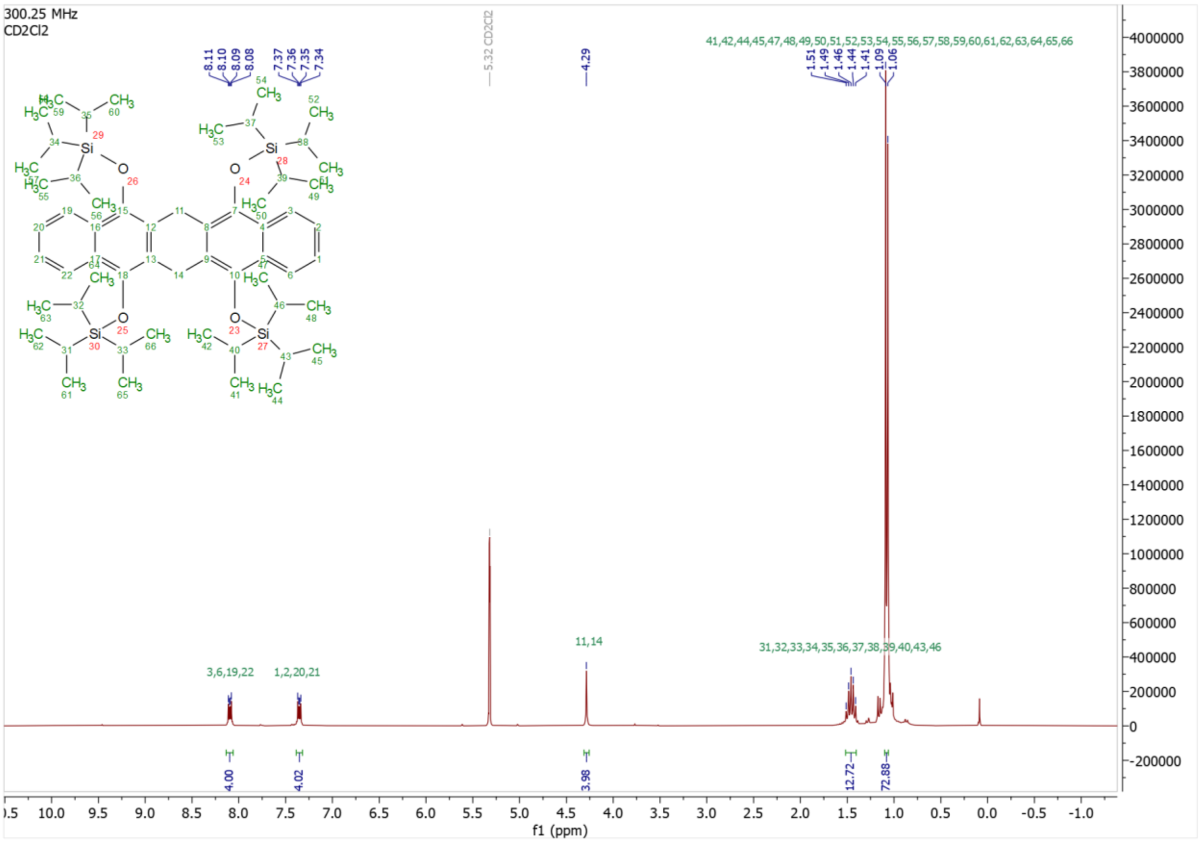

Figure S18: ^{1}H NMR (300 MHz, 298 K, CD_2Cl_2) of **10**.

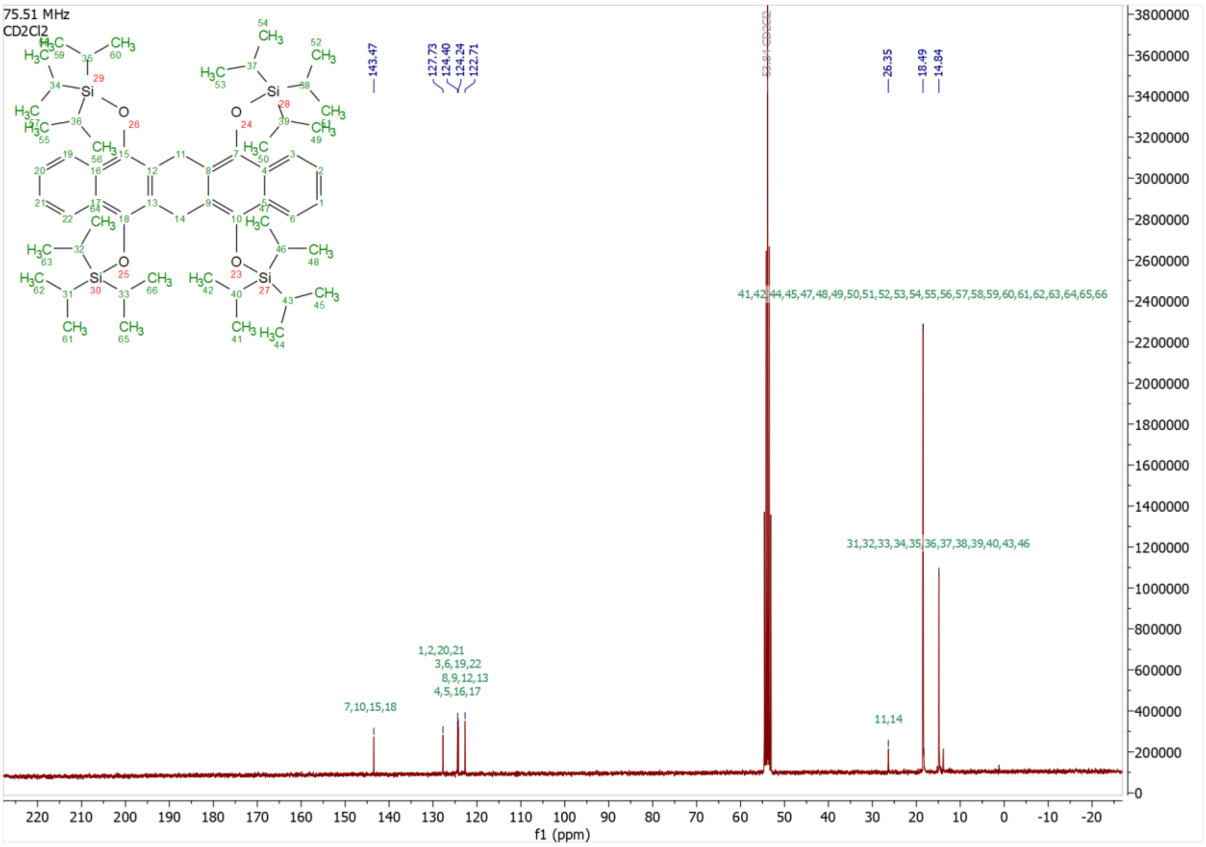

Figure S19: $^{13}C\{^1H\}$ NMR (300 MHz, 298 K, CD_2Cl_2) of **10**.

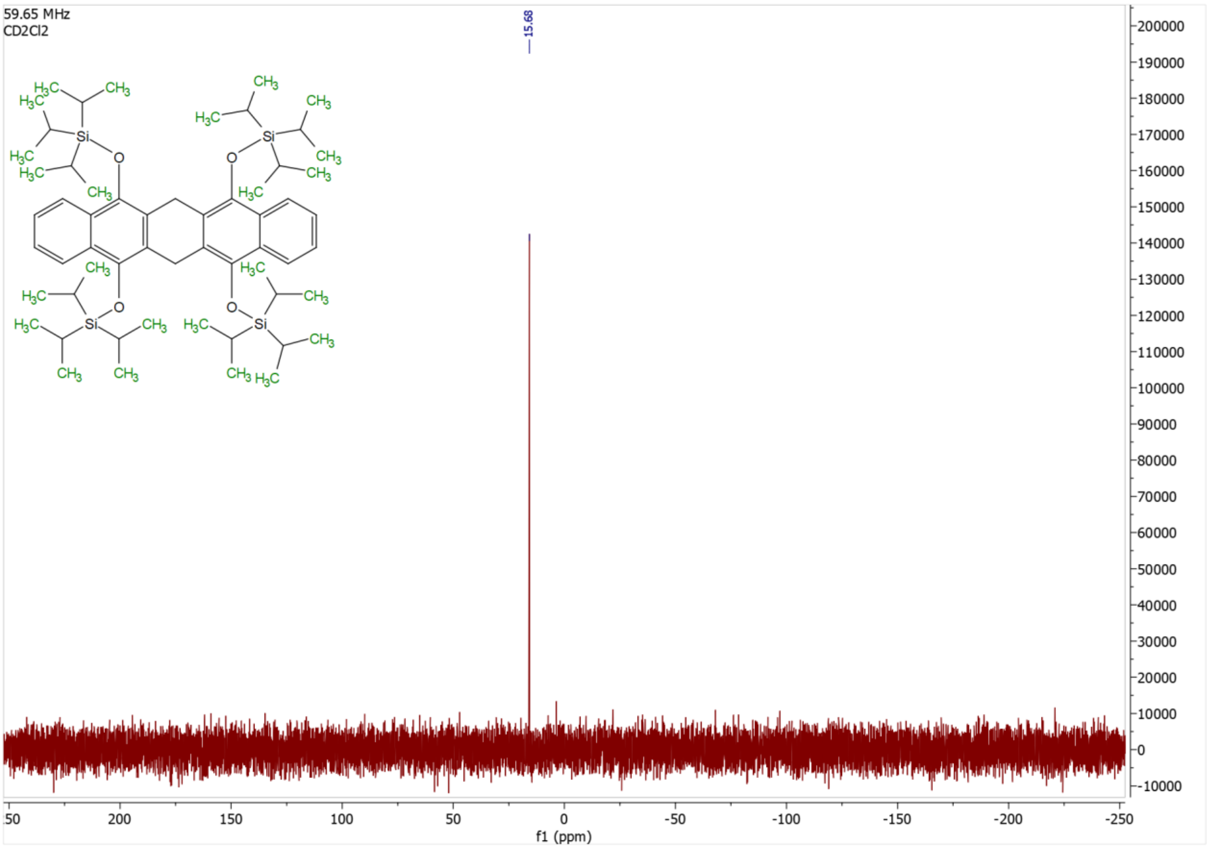

Figure S20: ^{29}Si NMR (300 MHz, 298 K, CD_2Cl_2) of **10**.

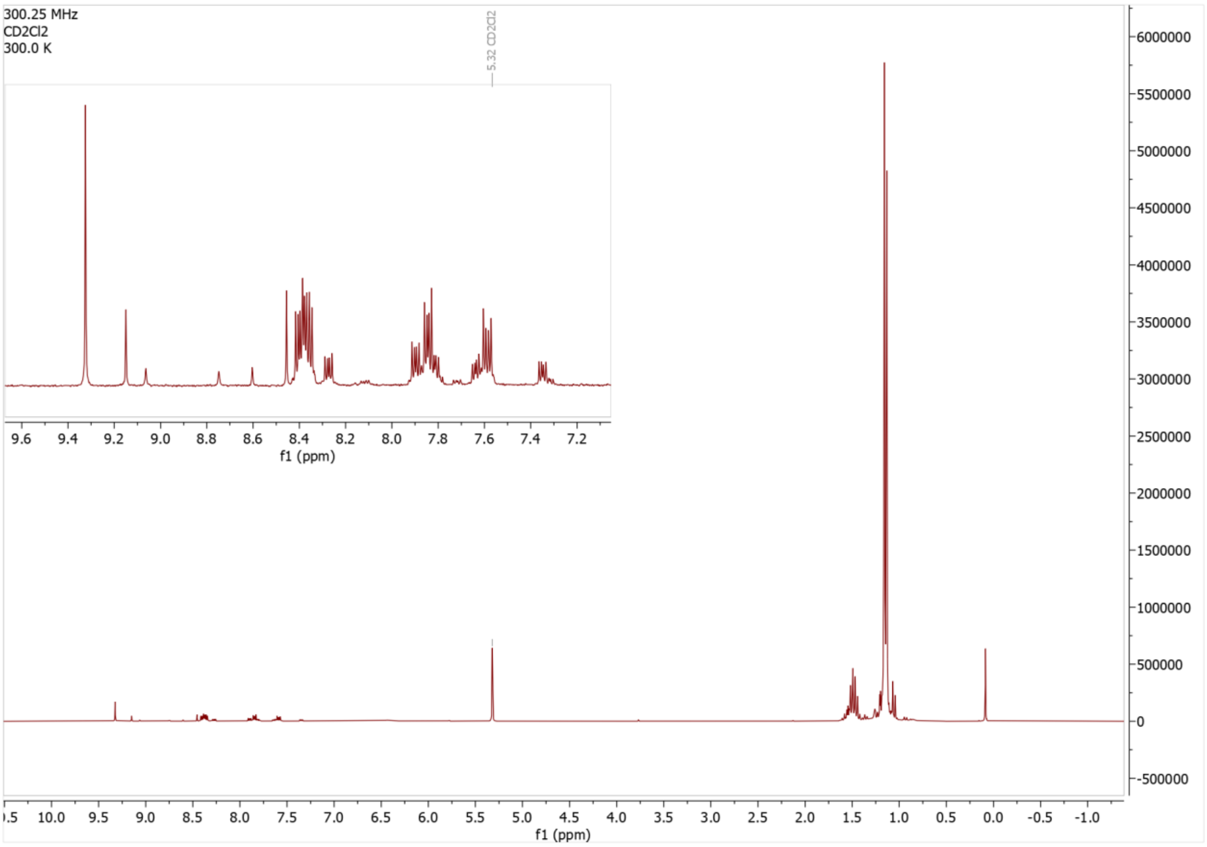

Figure S21: ¹H NMR of the reaction of **10** with 1.10 eq. of DDQ.

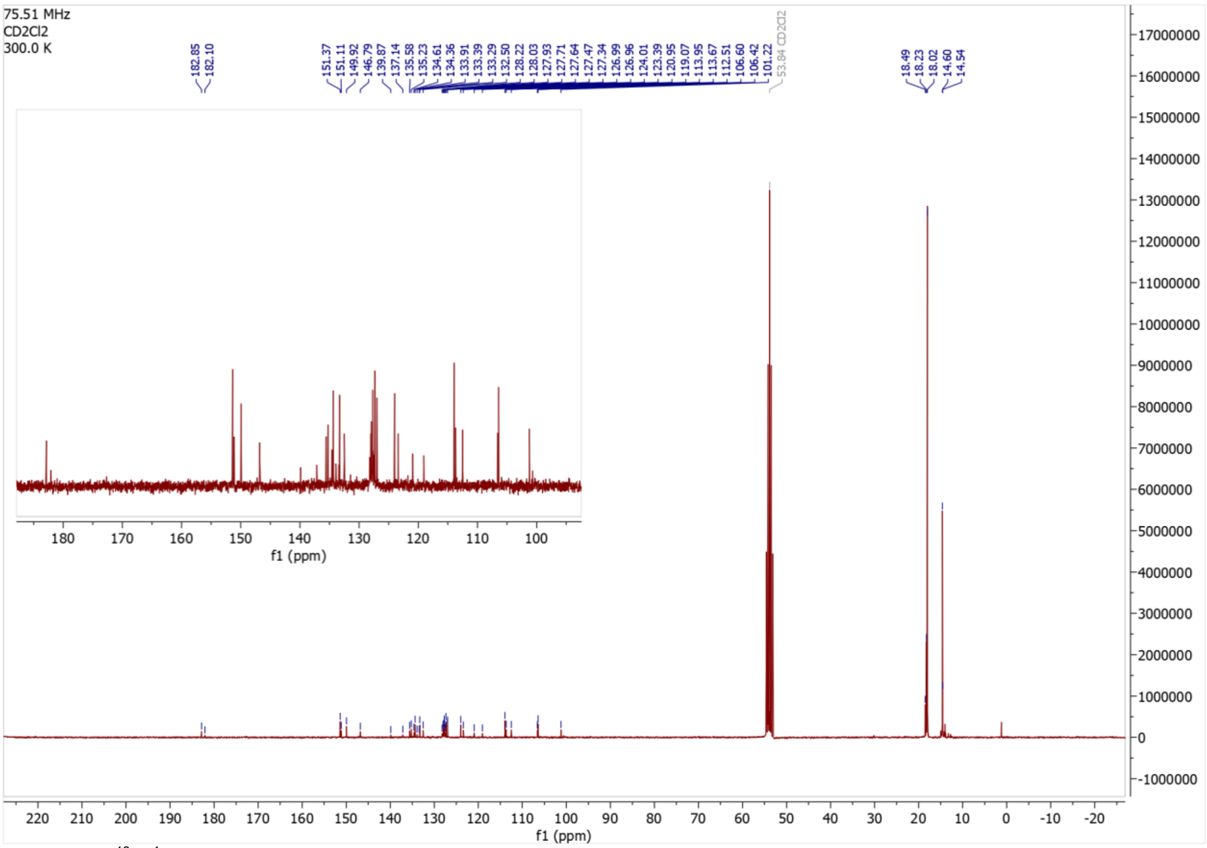

Figure S22: $^{13}C\{^1H\}$ NMR of the reaction of **10** with 1.10 eq. of DDQ.

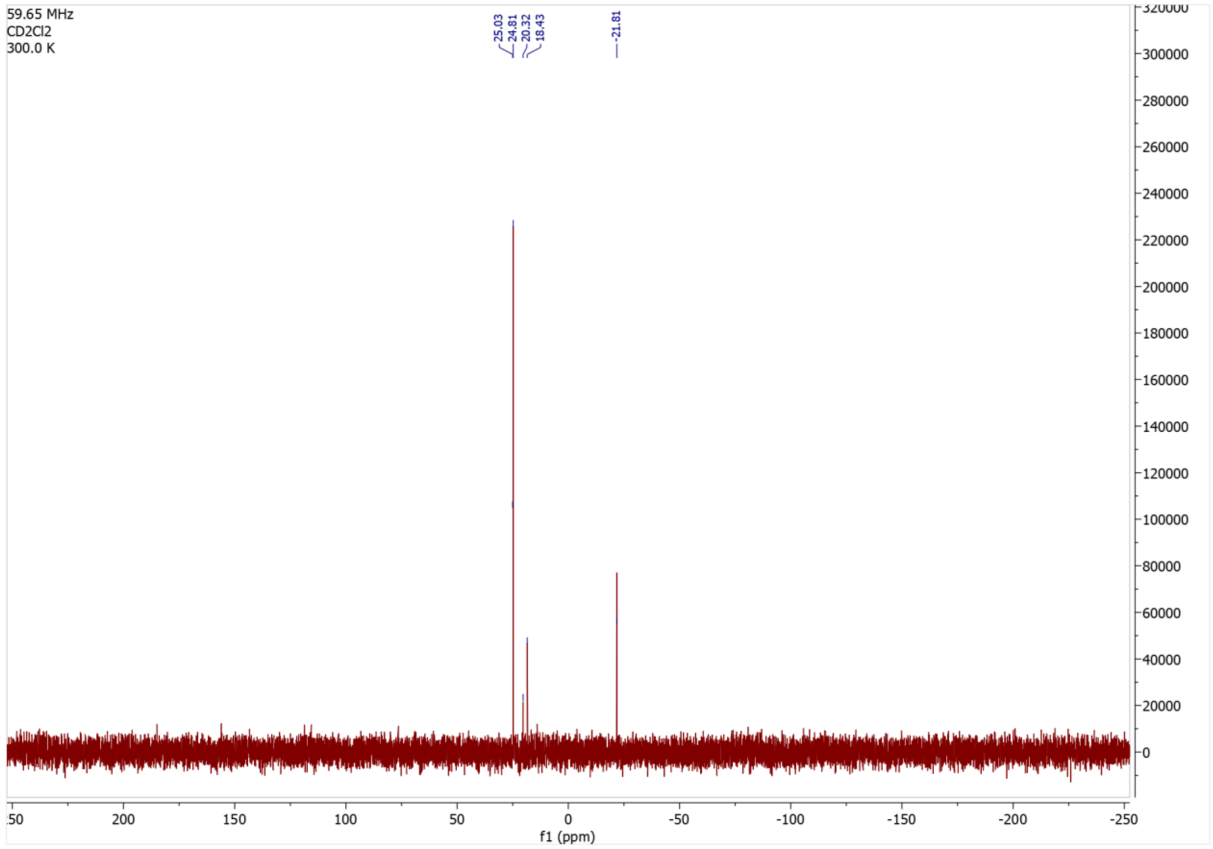

Figure S23: ^{29}Si NMR of the reaction of **10** with 1.10 eq. of DDQ.

Mass spectra

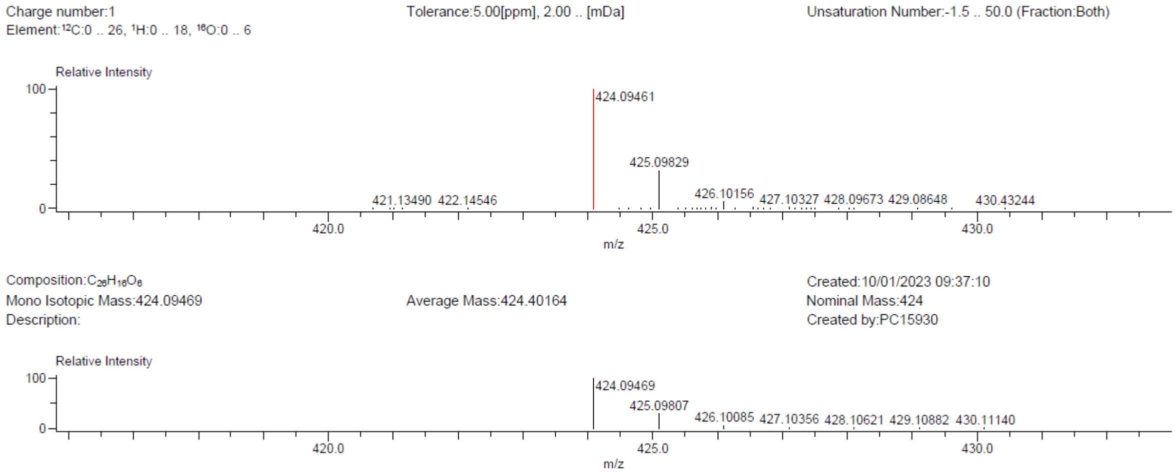

Mass	Intensity	Relative Intensity	Calc. Mass	Mass Difference [mDa]	Mass Difference [ppm]	Possible Formula	Unsaturation Number
424.09461	186019.45	100.00	424.09469	-0.08	-0.18	$^{12}C_{26}{}^{1}H_{16}{}^{16}O_6$	19.0

Figure S24: Mass spectrum (FD^+) of **7**.

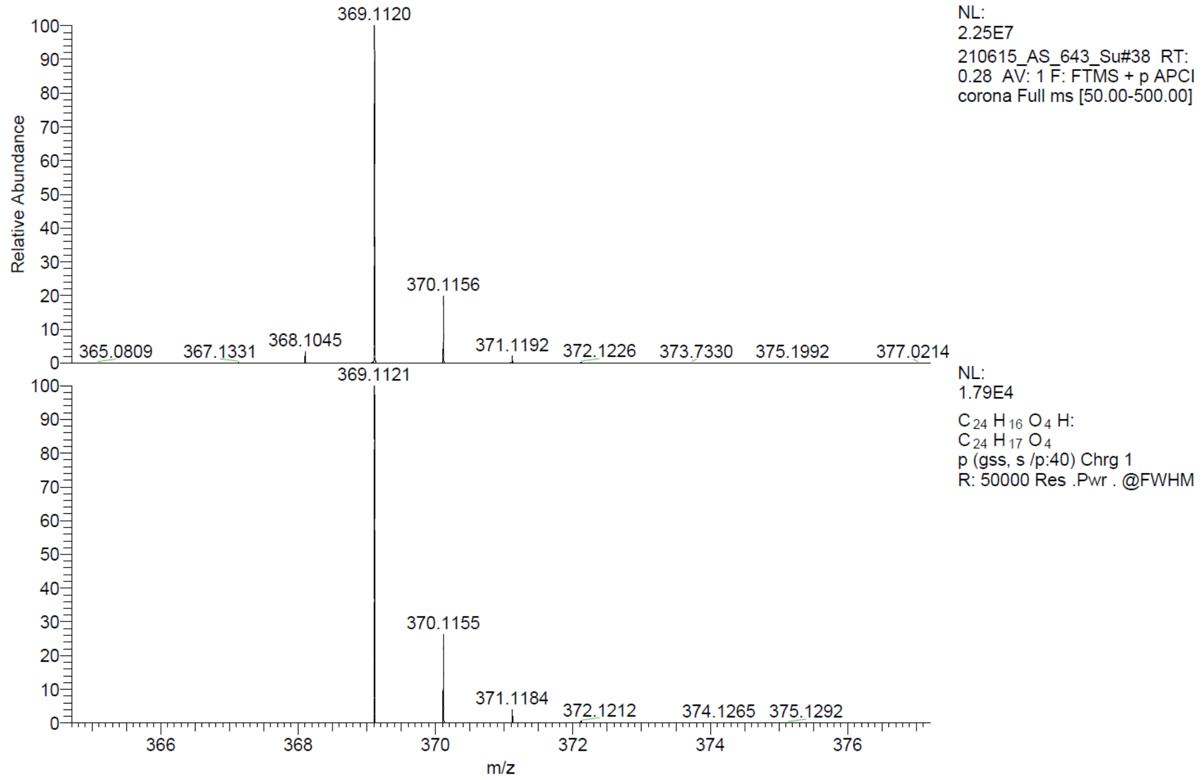

Figure S25: Mass spectrum (APCI$^+$) of **8**.

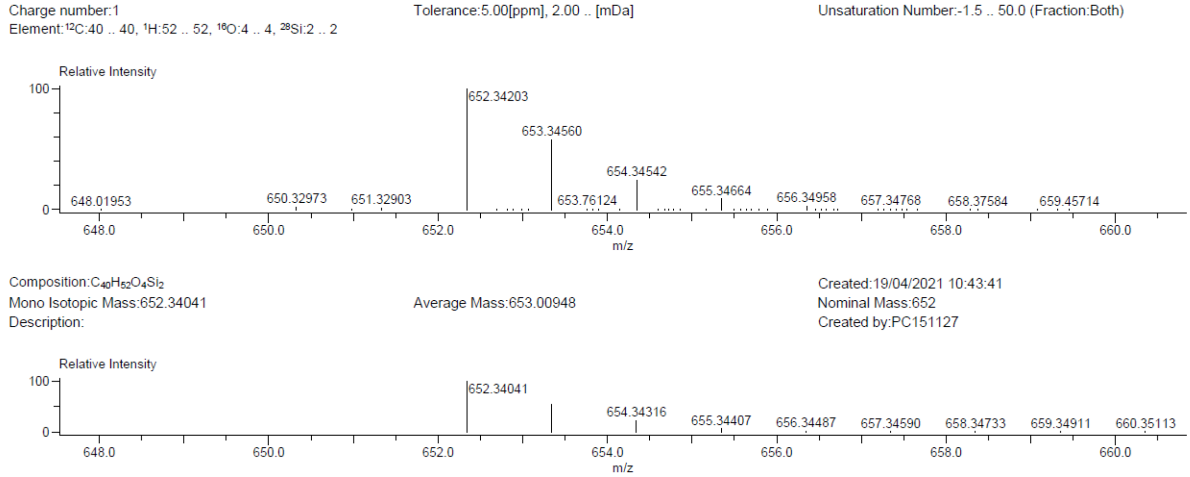

Mass	Intensity	Relative Intensity	Calc. Mass	Mass Difference [mDa]	Mass Difference [ppm]	Possible Formula	Unsaturation Number
652.34203	78714.18	100.00	652.34041	1.62	2.49	$^{12}C_{40}{}^{1}H_{52}{}^{16}O_4{}^{28}Si_2$	17.0

Figure S26: Mass spectrum (FD$^+$) of **9**.

Charge number:1
Element:^{12}C:58 .. 58, ^{1}H:96 .. 96, ^{16}O:4 .. 4, ^{28}Si:4 .. 4

Tolerance:5.00[ppm], 2.00 .. [mDa]

Unsaturation Number:-1.5 .. 50.0 (Fraction:Both)

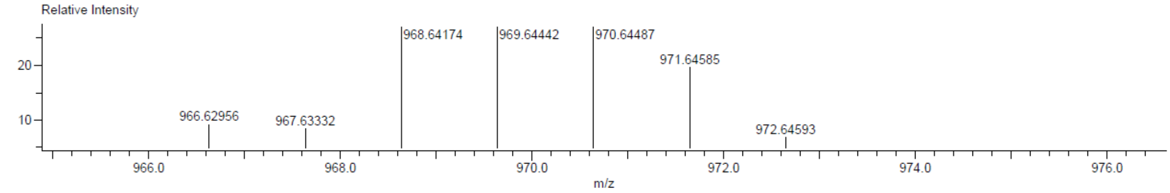

Composition:$C_{58}H_{96}O_4Si_4$
Mono Isotopic Mass:968.63856
Description:

Average Mass:969.72244

Created:31/05/2021 10:55:16
Nominal Mass:968
Created by:PC151127

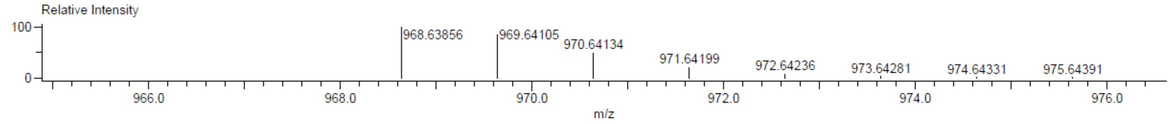

Mass	Intensity	Relative Intensity	Calc. Mass	Mass Difference [mDa]	Mass Difference [ppm]	Possible Formula	Unsaturation Number
968.64174	1353352.11	100.00	968.63856	3.18	3.28	$^{12}C_{58}{}^{1}H_{96}{}^{16}O_4{}^{28}Si_4$	15.0

Figure S27: Mass spectrum (FD$^+$) of **10**.

DFT and TD-DFT results

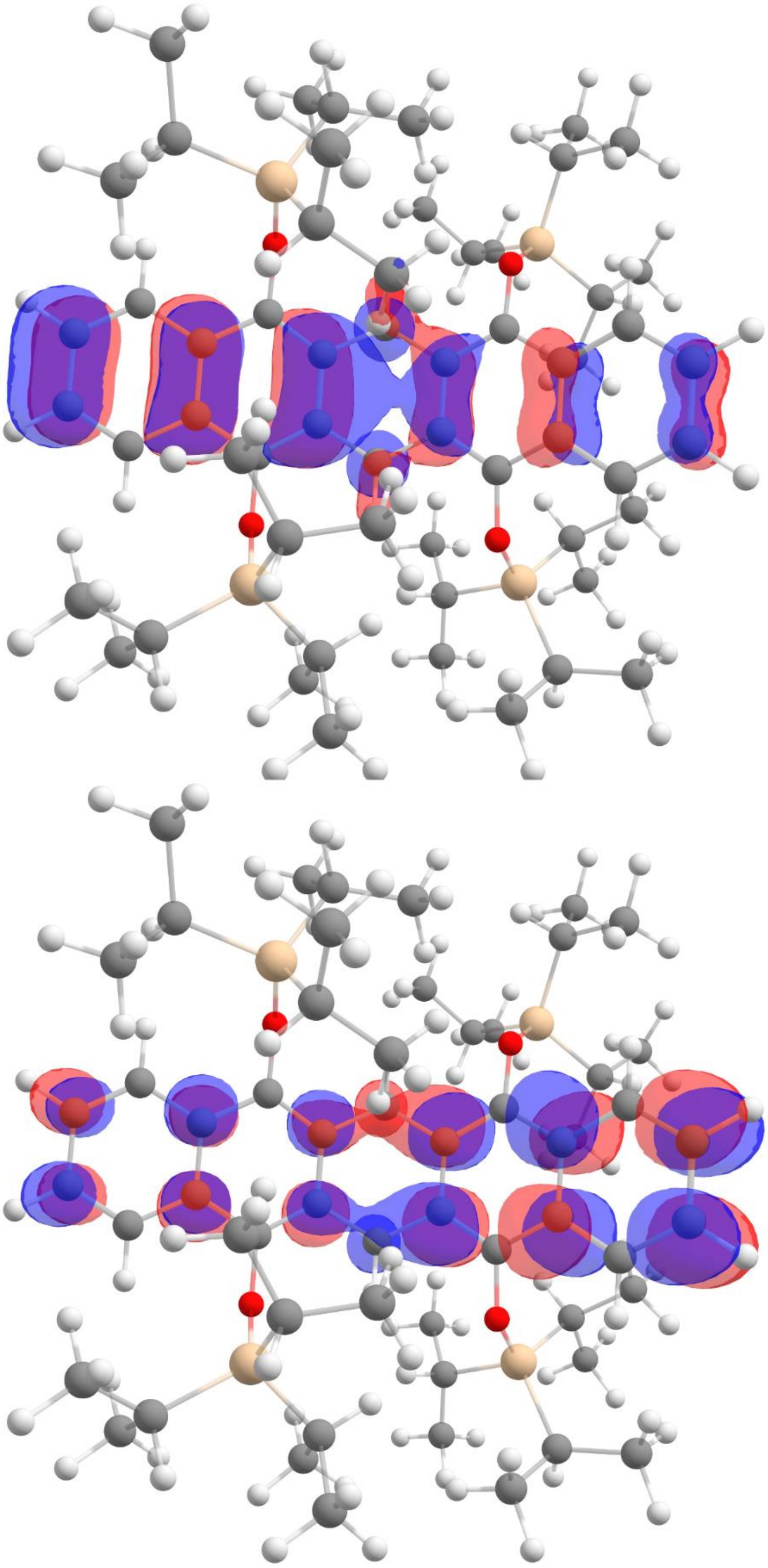

Figure S28: DFT-calculated Kohn-Sham molecular orbitals (HOMO-2 (top), LUMO+2 (bottom)) of **10** (B3LYP/def2-TZVPP level of theory, iso-value = 0.027). Homoconjugation between the two naphthalene moieties at the bridging sp^3 carbon atoms can be seen.

Table S6: Calculated main contribution transition energies and oscillator strengths derived from TD-DFT calculations in the UV and visible range.

Compound	Transition energy (wavelength)	Oscillator strength	Description of main contributors
**7**	4.690 eV<br>(264.3 nm)	1.522	HOMO-6 -> LUMO: 0.223062 (c= -0.47229392)<br>HOMO-2 -> LUMO: 0.141483 (c= -0.37614243)<br>HOMO-2 -> LUMO+1: 0.097152 (c= -0.31169208)<br>HOMO-1 -> LUMO+2: 0.411561 (c= -0.64153011)
	2.361 eV<br>(471.2 nm)	0.349	HOMO-2-> LUMO: 0.039288 (c= -0.19821284)<br>HOMO-1 -> HOMO: 0.942786 (c= 0.97097154)
**8**	4.094 eV<br>302.7 nm	0.914	HOMO-7 -> LUMO: 0.565348 (c= 0.75189606)<br>HOMO-4 -> HOMO: 0.100176 (c= -0.31650580)<br>HOMO-2 -> LUMO: 0.151519 (c= -0.38925415)<br>HOMO-1 -> LUMO+2: 0.083350 (c= 0.28870455)
	2.146 eV<br>577.7 nm	0.232	HOMO-2 -> LUMO: 0.056977 (c= 0.23869931)<br>HOMO-1 -> HOMO: 0.923536 (c= 0.96100796)
**9**	4.23 eV<br>286.9 nm	2.253	HOMO-7 -> LUMO: 0.110057 (c= -0.33174830)<br>HOMO-2 -> LUMO: 0.453201 (c= -0.67320178)<br>HUMO-1 -> LUMO+2: 0.294127 (c= -0.54233479)
	2.27 eV<br>545 nm	0.423	HOMO-2 -> LUMO: 0.025649 (c= 0.16015191)<br>HOMO-1 -> HOMO: 0.957014 (c= -0.97827079)
**10**	5.15 eV<br>240.7 nm	2.000	HOMO-4 -> HOMO: 0.178433 (c= -0.42241358)<br>HOMO-3 -> LUMO: 0.407227 (c= -0.63814329)
	3.97 eV<br>312.3 nm	0.165	HOMO-2 -> LUMO 0.932224 (c= 0.96551749)

Cartesian coordinates (XYZ) of DFT optimized geometry of **7** (B3LYP/def2-TZVPP).

Symmetry: c1

```
C    0.599410000   -5.921338000   -0.471127000
C   -0.816759000   -5.884795000   -0.389070000
C   -1.474156000   -4.700383000   -0.223962000
C   -0.749950000   -3.479162000   -0.129654000
C    0.686321000   -3.516520000   -0.211588000
C    1.331319000   -4.772654000   -0.385826000
C   -1.381330000   -2.245402000    0.039498000
C   -0.671680000   -1.049090000    0.132090000
C    0.765168000   -1.087467000    0.057496000
C    1.396214000   -2.318148000   -0.115134000
C   -1.310182000    0.204846000    0.297717000
C   -0.589253000    1.363840000    0.394701000
C    0.840881000    1.324245000    0.330974000
C    1.484603000    0.128516000    0.164436000
C   -1.311234000    2.647635000    0.566565000
C   -0.492459000    3.881780000    0.682324000
C    0.910858000    3.842057000    0.625889000
C    1.646278000    2.564184000    0.445679000
C   -1.140037000    5.106374000    0.851684000
C   -0.408056000    6.277526000    0.962961000
C    0.984766000    6.238138000    0.906705000
C    1.638092000    5.027886000    0.739443000
O    2.867863000    2.539632000    0.394520000
O   -2.532434000    2.692712000    0.612048000
O    2.782963000   -2.363225000   -0.130442000
O   -2.768405000   -2.202898000    0.055964000
C   -3.404299000   -2.285941000    1.270138000
C   -4.884821000   -2.216274000    1.089158000
O   -2.805846000   -2.397665000    2.305086000
C    3.422514000   -2.201871000   -1.334302000
O    2.826216000   -2.032072000   -2.362471000
C    4.902903000   -2.269624000   -1.151638000
H    1.099236000   -6.871523000   -0.602755000
H   -1.376491000   -6.807670000   -0.459275000
H   -2.552469000   -4.677457000   -0.165998000
H    2.409269000   -4.806059000   -0.445607000
H   -2.387306000    0.256886000    0.346188000
H    2.563100000    0.120267000    0.119630000
H   -2.219468000    5.122634000    0.894116000
H   -0.917999000    7.222489000    1.093956000
```

H	1.556168000	7.152497000	0.993509000
H	2.716598000	4.983292000	0.693888000
H	-5.377145000	-2.284109000	2.054628000
H	-5.152106000	-1.279140000	0.599175000
H	-5.215956000	-3.031106000	0.444047000
H	5.225959000	-1.467911000	-0.485985000
H	5.175342000	-3.216108000	-0.683458000
H	5.397496000	-2.172520000	-2.113421000

Final Gibbs free energy: −1451.40445161 Eh

Cartesian coordinates (XYZ) of DFT optimized geometry of **8** (B3LYP /def2-TZVPP)

symmetry c1

C	0.849414000	6.035815000	1.504842000
C	-0.545024000	6.074326000	1.464686000
C	-1.266803000	4.908685000	1.263131000
C	-0.607562000	3.690035000	1.091079000
C	0.796131000	3.650317000	1.144462000
C	1.513508000	4.829665000	1.351832000
C	-1.414302000	2.465915000	0.843235000
C	-0.678811000	1.199479000	0.628736000
C	0.750832000	1.154817000	0.727154000
C	1.542508000	2.376549000	0.988869000
C	-1.377806000	0.058641000	0.332718000
C	-0.721406000	-1.182587000	0.134804000
C	0.709273000	-1.235819000	0.292762000
C	1.407066000	-0.035548000	0.570424000
C	-1.418366000	-2.350871000	-0.193745000
C	-0.747766000	-3.576964000	-0.332319000
C	0.680511000	-3.628661000	-0.172499000
C	1.379636000	-2.455141000	0.146521000
C	-1.449291000	-4.784019000	-0.608245000
C	-0.777664000	-5.965526000	-0.740833000
C	0.636151000	-6.009487000	-0.620790000
C	1.345179000	-4.875130000	-0.345987000
O	2.739873000	-2.495792000	0.295363000
C	3.188033000	-2.613403000	1.657034000
O	-2.776811000	-2.306149000	-0.356376000
C	-3.206689000	-2.192898000	-1.724776000
O	2.763938000	2.346256000	1.071303000
O	-2.637266000	2.512486000	0.812958000
H	1.412867000	6.945559000	1.662022000
H	-1.063984000	7.014570000	1.592996000
H	-2.346542000	4.924967000	1.229577000
H	2.592202000	4.781191000	1.390828000
H	-2.453731000	0.104336000	0.256058000
H	2.483357000	-0.052107000	0.648018000
H	-2.524855000	-4.750535000	-0.701105000
H	-1.323876000	-6.876714000	-0.944757000
H	1.151329000	-6.951916000	-0.749553000
H	2.421197000	-4.904040000	-0.256061000
H	2.741301000	-1.840141000	2.283037000
H	4.268154000	-2.488971000	1.642546000
H	2.932620000	-3.594696000	2.063255000
H	-2.976602000	-1.199739000	-2.117749000
H	-2.724583000	-2.945315000	-2.349536000
H	-4.282442000	-2.352739000	-1.730721000

Final Gibbs free energy: −1224.69197680 Eh

Cartesian coordinates (XYZ) of DFT optimized geometry of **9** (B3LYP/def2-TZVPP). *^i^*Pr groups are simplified as Me groups.

Symmetry: c1

C	0.598505000	-5.932272000	-1.552579000
C	-0.729679000	-6.038816000	-1.071197000
C	-1.362170000	-4.950180000	-0.539573000
C	-0.704357000	-3.693095000	-0.451780000
C	0.653302000	-3.592540000	-0.913075000
C	1.269917000	-4.744510000	-1.472635000

C	-1.349160000	-2.560984000	0.080889000
C	-0.664844000	-1.342970000	0.185996000
C	0.693282000	-1.238829000	-0.288977000
C	1.337771000	-2.366689000	-0.813669000
C	-1.267413000	-0.196821000	0.758556000
C	-0.610286000	1.002154000	0.841516000
C	0.724422000	1.116916000	0.328884000
C	1.338254000	0.019296000	-0.213171000
C	-1.294514000	2.158512000	1.456308000
C	-0.546482000	3.442703000	1.509902000
C	0.758195000	3.556896000	1.000720000
C	1.456463000	2.399498000	0.381195000
C	-1.160879000	4.557387000	2.083051000
C	-0.493165000	5.769360000	2.153214000
C	0.802303000	5.882321000	1.648882000
C	1.421436000	4.782928000	1.076658000
O	2.610784000	-2.270894000	-1.263754000
Si	4.054817000	-2.380417000	-0.390881000
C	5.169757000	-1.097272000	-1.149592000
C	3.706845000	-2.051839000	1.411910000
C	4.763934000	-4.092728000	-0.608354000
O	-2.623654000	-2.663327000	0.525044000
Si	-4.059714000	-2.280469000	-0.282056000
C	-5.113091000	-1.392360000	0.970505000
C	-3.658053000	-1.212472000	-1.757349000
C	-4.875723000	-3.870588000	-0.817474000
O	2.593116000	2.514792000	-0.061395000
O	-2.430822000	2.073922000	1.906403000
H	1.081748000	-6.798884000	-1.983792000
H	-1.244282000	-6.988827000	-1.127847000
H	-2.372419000	-5.032404000	-0.170306000
H	2.280359000	-4.662865000	-1.841418000
H	-2.269429000	-0.264625000	1.152455000
H	2.340425000	0.125833000	-0.597885000
H	-2.164353000	4.455343000	2.470219000
H	-0.976956000	6.627696000	2.599812000
H	1.324503000	6.828153000	1.703957000
H	2.424583000	4.855567000	0.681862000
H	6.164249000	-1.134184000	-0.698039000
H	5.285566000	-1.264959000	-2.223039000
H	4.772824000	-0.090309000	-1.005278000
H	4.626630000	-2.184535000	1.987994000
H	2.966511000	-2.749819000	1.809510000
H	3.347470000	-1.037186000	1.589088000
H	4.132022000	-4.852977000	-0.145364000
H	4.891451000	-4.347980000	-1.662597000
H	5.747944000	-4.146243000	-0.134067000
H	-6.106243000	-1.191243000	0.560833000
H	-5.241745000	-1.995363000	1.872551000
H	-4.672023000	-0.437101000	1.261735000
H	-4.571969000	-1.011564000	-2.322694000
H	-2.955799000	-1.708290000	-2.431334000
H	-3.229916000	-0.251765000	-1.466637000
H	-4.297993000	-4.386127000	-1.586641000
H	-5.010619000	-4.552829000	0.024822000
H	-5.864773000	-3.657544000	-1.232532000

Final Gibbs free energy: −1963.34388252 Eh

Cartesian coordinates (XYZ) of DFT optimized geometry of **10** (B3LYP/def2-TZVPP).

Symmetry: c1

C	-1.223126000	-0.960383000	5.633603000
C	-1.175605000	0.445658000	5.716649000
C	-0.601893000	1.177240000	4.708712000
C	-0.051647000	0.542934000	3.571081000
C	-0.077875000	-0.879746000	3.499577000
C	-0.682318000	-1.604053000	4.551467000
C	0.495950000	1.271811000	2.481958000
C	0.783475000	0.625774000	1.302456000
C	0.794891000	-0.791583000	1.242088000
C	0.483219000	-1.527809000	2.364918000

C	1.057388000	1.389404000	0.035374000
C	0.443963000	0.725411000	-1.169285000
C	0.516506000	-0.689995000	-1.247663000
C	1.131110000	-1.428979000	-0.083347000
C	-0.167744000	1.450156000	-2.170336000
C	-0.467202000	0.815208000	-3.407608000
C	-0.376593000	-0.604717000	-3.495411000
C	0.021505000	-1.346734000	-2.351324000
C	-0.797684000	1.543803000	-4.572122000
C	-1.066582000	0.907969000	-5.756860000
C	-1.016365000	-0.496589000	-5.831776000
C	-0.673638000	-1.230100000	-4.724865000
O	0.604825000	-2.888830000	2.390397000
O	-0.055747000	-2.713324000	-2.388754000
O	-0.421076000	2.786220000	-2.016235000
O	0.666846000	2.625846000	2.602230000
Si	2.056115000	3.383110000	3.201721000
C	2.719335000	2.331679000	4.634727000
C	1.392922000	5.080334000	3.697520000
C	3.333627000	3.504602000	1.805041000
C	3.599472000	3.101276000	5.630930000
C	3.434291000	1.058808000	4.150541000
C	2.465058000	6.121479000	4.046792000
C	0.326957000	4.992155000	4.797872000
C	2.884565000	4.435634000	0.670815000
C	4.750842000	3.880673000	2.265997000
Si	1.890823000	-3.866117000	2.868000000
C	3.094266000	-3.014290000	4.061635000
C	2.830198000	-4.327175000	1.277051000
C	1.096984000	-5.420679000	3.597030000
C	4.161084000	-4.043308000	4.482876000
C	2.527746000	-2.317414000	5.304265000
C	3.368740000	-5.763775000	1.220649000
C	3.952242000	-3.335965000	0.930762000
C	-0.015863000	-5.938170000	2.676929000
C	0.589758000	-5.315601000	5.040174000
Si	-1.390559000	-3.666653000	-2.006269000
C	-0.719126000	-4.998733000	-0.842588000
C	-2.106204000	-4.523138000	-3.546959000
C	-2.742078000	-2.686870000	-1.102161000
C	0.515586000	-5.739280000	-1.372061000
C	-1.809355000	-5.994435000	-0.422696000
C	-3.215179000	-3.772727000	-4.298747000
C	-1.043464000	-5.041143000	-4.529188000
C	-3.184925000	-1.367994000	-1.758740000
C	-2.437907000	-2.432211000	0.382402000
Si	-1.869844000	3.584598000	-1.670034000
C	-3.048419000	2.483352000	-0.676827000
C	-1.339044000	5.158232000	-0.769413000
C	-2.720745000	4.102893000	-3.299520000
C	-2.451534000	1.741575000	0.524944000
C	-4.281196000	3.297870000	-0.244967000
C	-0.167030000	5.825898000	-1.506536000
C	-1.025156000	4.989307000	0.719630000
C	-3.426369000	5.467853000	-3.235088000
C	-3.691477000	3.045112000	-3.847972000
H	-1.693205000	-1.531136000	6.423287000
H	-1.606578000	0.949669000	6.571377000
H	-0.588409000	2.254281000	4.756687000
H	-0.722499000	-2.678811000	4.475721000
H	0.700568000	2.407002000	0.129887000
H	2.138408000	1.460164000	-0.139898000
H	2.217177000	-1.431141000	-0.227778000
H	0.826886000	-2.469170000	-0.089090000
H	-0.805369000	2.619552000	-4.520026000
H	-1.305784000	1.487801000	-6.638266000
H	-1.225205000	-0.996154000	-6.768450000
H	-0.590813000	-2.301351000	-4.785083000
H	1.828551000	2.007211000	5.179535000
H	0.889671000	5.428255000	2.789912000
H	3.387606000	2.487498000	1.401028000
H	4.492320000	3.514628000	5.160108000
H	3.062423000	3.925819000	6.099009000

H	3.935376000	2.435216000	6.430333000
H	4.348257000	1.292267000	3.603242000
H	2.805826000	0.454806000	3.497259000
H	3.716022000	0.435425000	5.001632000
H	3.050545000	5.833381000	4.920242000
H	3.161057000	6.285571000	3.224795000
H	1.997568000	7.083254000	4.273975000
H	-0.511781000	4.369734000	4.488622000
H	-0.067965000	5.984363000	5.032031000
H	0.732340000	4.578714000	5.723921000
H	1.907211000	4.169539000	0.277180000
H	3.593214000	4.400902000	-0.160791000
H	2.829987000	5.472917000	1.005949000
H	5.149926000	3.182753000	2.999703000
H	5.435714000	3.886527000	1.413823000
H	4.782181000	4.876318000	2.709709000
H	3.598967000	-2.247121000	3.465091000
H	2.066247000	-4.243406000	0.499618000
H	1.902696000	-6.163352000	3.592534000
H	4.941232000	-3.560148000	5.076061000
H	3.726882000	-4.831744000	5.100744000
H	4.645990000	-4.523538000	3.633555000
H	3.347837000	-1.941646000	5.922696000
H	1.940798000	-2.993597000	5.923982000
H	1.893816000	-1.473904000	5.051651000
H	2.583572000	-6.506040000	1.357774000
H	3.834842000	-5.957653000	0.250998000
H	4.127644000	-5.944057000	1.984185000
H	4.298152000	-3.486094000	-0.094896000
H	3.640538000	-2.296224000	1.024300000
H	4.813543000	-3.473446000	1.586453000
H	0.339429000	-6.110066000	1.662350000
H	-0.422210000	-6.882150000	3.049571000
H	-0.837592000	-5.223433000	2.616147000
H	0.160379000	-6.269138000	5.359729000
H	1.383553000	-5.063113000	5.741347000
H	-0.192855000	-4.563249000	5.142270000
H	-0.411287000	-4.448266000	0.052291000
H	-2.581430000	-5.407657000	-3.104183000
H	-3.595239000	-3.377797000	-1.149260000
H	0.964566000	-6.355764000	-0.589488000
H	0.253042000	-6.411115000	-2.189636000
H	1.279728000	-5.052143000	-1.736161000
H	-1.431130000	-6.702203000	0.317435000
H	-2.158940000	-6.580471000	-1.274829000
H	-2.677763000	-5.500217000	0.015449000
H	-3.614228000	-4.397953000	-5.102502000
H	-2.850293000	-2.852596000	-4.754447000
H	-4.047033000	-3.510788000	-3.645330000
H	-0.512357000	-4.226475000	-5.020595000
H	-0.292982000	-5.661007000	-4.043202000
H	-1.511908000	-5.640941000	-5.314121000
H	-3.360897000	-1.454253000	-2.828817000
H	-4.108289000	-1.010330000	-1.295321000
H	-2.432540000	-0.595491000	-1.614501000
H	-1.624451000	-1.718339000	0.500111000
H	-3.311026000	-2.004419000	0.881661000
H	-2.158464000	-3.334574000	0.923928000
H	-3.392328000	1.721814000	-1.384722000
H	-2.201728000	5.828156000	-0.843017000
H	-1.900583000	4.211848000	-4.016159000
H	-2.008240000	2.416109000	1.255771000
H	-3.229213000	1.170646000	1.039353000
H	-1.684290000	1.035632000	0.222310000
H	-4.010101000	4.083973000	0.462164000
H	-4.787942000	3.772225000	-1.085163000
H	-5.007768000	2.651588000	0.254023000
H	0.095993000	6.776073000	-1.034074000
H	-0.400295000	6.030253000	-2.552997000
H	0.717599000	5.189697000	-1.491510000
H	-1.899302000	4.676940000	1.289706000
H	-0.679434000	5.936011000	1.144673000
H	-0.249291000	4.248896000	0.899707000

H	-2.737868000	6.279581000	-3.003984000
H	-4.217122000	5.483470000	-2.482896000
H	-3.893274000	5.696824000	-4.196814000
H	-3.248624000	2.052551000	-3.902563000
H	-4.584209000	2.975609000	-3.224368000
H	-4.021463000	3.311764000	-4.855430000

Final Gibbs free energy: -3725.41157472 Eh

[1] W. L. F. Armarego, D. D. Perrin, *Purification of Laboratory Chemicals*, Butterworth Heinemann, **1997**.
[2] G. R. Fulmer, A. J. M. Miller, N. H. Sherden, H. E. Gottlieb, A. Nudelman, B. M. Stoltz, J. E. Bercaw, K. I. Goldberg, *Organometallics* **2010**, *29*, 2176–2179.
[3] G. M. Sheldrick, *Acta Crystallogr., Sect. C: Struct. Chem.* **2015**, *71*, 3–8.
[4] C. B. Hübschle, G. M. Sheldrick, B. Dittrich, *J. Appl. Crystallogr.* **2011**, *44*, 1281–1284.
[5] A. L. Spek, *Acta Crystallogr., Sect. D: Biol. Crystallogr.* **2009**, *65*, 148–155.
[6] A. D. Becke, *J. Phys. Chem.* **1993**, *98*, 1372–1377.
[7] C. Lee, E. Yang, R. G. Parr, *Phys. Rev. B* **1988**, *37*, 785–789.
[8] P. J. Stephens, F. J. Devlin, C. F. Chabalowski, M. J. Frisch, *J. Phys. Chem.* **1994**, *98*, 11623–11627.
[9] L. Wilk A N, D. M. Nusair, *Can. J. Phys.* **1980**, *58*, 1200.
[10] F. Weigend, R. Ahlrichs, *Phys. Chem. Chem. Phys.* **2005**, *7*, 3297–3305.
[11] V. Barone, M. Cossi, *J. Phys. Chem. A* **1998**, *102*, 1995–2001.
[12] F. Neese, F. Wennmohs, U. Becker, C. Riplinger, *J. Chem. Phys.* **2020**, *152*, 224108.
[13] F. Weigend, M. Haser, H. Patzelt, R. Ahlrichs, *Chem. Phys. Lett.* **1998**, *294*, 143–152.
[14] S. Grimme, *Chem. - Eur. J.* **2012**, *18*, 9955–9964.
[15] G. Herzberg, *Infrared and Raman Spectra of Polyatomic Molecules*, Van Nostrand Reinhold, **1945**.
[16] C. C. Mattheus, J. Baas, A. Meetsma, J. L. de Boer, C. Kloc, T. Siegrist, T. T. M. Palstra, *Acta Crystallogr., Sect. E: Struct. Rep. Online* **2002**, *58*, o1229–o1231.
[17] T. Tajima, R. Sanda, K. Nishihara, H. Shirai, Y. Okuda, A. Orita, Y. Takaguchi, *RSC Adv.* **2019**, *9*, 17035–17039.
[18] D. Lehnherr, A. H. Murray, R. McDonald, R. R. Tykwinski, *Angew. Chem., Int. Ed.* **2010**, *49*, 6190–6194.
[19] S. Grimme, J. Antony, S. Ehrlich, H. Krieg, *J. Chem. Phys.* **2010**, *132*.
[20] A. D. Becke, *J. Chem. Phys.* **1993**, *98*, 5648–5652.
[21] Gaussian 16, Revision C.01, Frisch, M. J.; Trucks, G. W.; Schlegel, H. B.; Scuseria, G. E.; Robb, M. A.; Cheeseman, J. R.; Scalmani, G.; Barone, V.; Petersson, G. A.; Nakatsuji, H.; Li, X.; Caricato, M.; Marenich, A. V.; Bloino, J.; Janesko, B. G.; Gomperts, R.; Mennucci, B.; Hratchian, H. P.; Ortiz, J. V.; Izmaylov, A. F.; Sonnenberg, J. L.; Williams-Young, D.; Ding, F.; Lipparini, F.; Egidi, F.; Goings, J.; Peng, B.; Petrone, A.; Henderson, T.; Ranasinghe, D.; Zakrzewski, V. G.; Gao, J.; Rega, N.; Zheng, G.; Liang, W.; Hada, M.; Ehara, M.; Toyota, K.; Fukuda, R.; Hasegawa, J.; Ishida, M.; Nakajima, T.; Honda, Y.; Kitao, O.; Nakai, H.; Vreven, T.; Throssell, K.; Montgomery, J. A., Jr.; Peralta, J. E.; Ogliaro, F.; Bearpark, M. J.; Heyd, J. J.; Brothers, E. N.; Kudin, K. N.; Staroverov, V. N.; Keith, T. A.; Kobayashi, R.; Normand, J.; Raghavachari, K.; Rendell, A. P.; Burant, J. C.; Iyengar, S. S.; Tomasi, J.; Cossi, M.; Millam, J. M.; Klene, M.; Adamo, C.; Cammi, R.; Ochterski, J. W.; Martin, R. L.; Morokuma, K.; Farkas, O.; Foresman, J. B.; Fox, D. J. Gaussian, Inc., Wallingford CT, **2016**.
[22] H. Günther, in *NMR Spectroscopy, Basic Principles, Concepts, and Applications in Chemistry*, Wiley-VCH, Weinheim, **2013**.
